# A double-network porous hydrogel based on high internal phase emulsions as a vehicle for potassium sucrose octasulfate delivery accelerates diabetic wound healing

**DOI:** 10.1093/rb/rbae024

**Published:** 2024-03-12

**Authors:** Zhiwei Wang, Lingshun Sun, Weixing Wang, Zheng Wang, Ge Shi, Honglian Dai, Aixi Yu

**Affiliations:** Department of Orthopedics Trauma and Microsurgery, Zhongnan Hospital of Wuhan University, Wuhan 430070, China; State Key Laboratory of Advanced Technology for Materials Synthesis and Processing, Biomedical Materials and Engineering Research Center of Hubei Province, Wuhan University of Technology, Wuhan 430070, China; Department of Orthopedics Trauma and Microsurgery, Zhongnan Hospital of Wuhan University, Wuhan 430070, China; Department of Orthopedics Trauma and Microsurgery, Zhongnan Hospital of Wuhan University, Wuhan 430070, China; Department of Orthopedics Trauma and Microsurgery, Zhongnan Hospital of Wuhan University, Wuhan 430070, China; State Key Laboratory of Advanced Technology for Materials Synthesis and Processing, Biomedical Materials and Engineering Research Center of Hubei Province, Wuhan University of Technology, Wuhan 430070, China; Department of Orthopedics Trauma and Microsurgery, Zhongnan Hospital of Wuhan University, Wuhan 430070, China

**Keywords:** high internal phase emulsions, double-network porous hydrogel, drug delivery, diabetic wound healing, sucrose octasulfate

## Abstract

Diabetic wounds are a difficult medical challenge. Excessive secretion of matrix metalloproteinase-9 (MMP-9) in diabetic wounds further degrades the extracellular matrix and growth factors and causes severe vascular damage, which seriously hinders diabetic wound healing. To solve these issues, a double-network porous hydrogel composed of poly (methyl methacrylate-co-acrylamide) (p(MMA-co-AM)) and polyvinyl alcohol (PVA) was constructed by the high internal phase emulsion (HIPE) technique for the delivery of potassium sucrose octasulfate (PSO), a drug that can inhibit MMPs, increase angiogenesis and improve microcirculation. The hydrogel possessed a typical polyHIPE hierarchical microstructure with interconnected porous morphologies, high porosity, high specific surface area, excellent mechanical properties and suitable swelling properties. Meanwhile, the p(MMA-co-AM)/PVA@PSO hydrogel showed high drug-loading performance and effective PSO release. In addition, both *in vitro* and *in vivo* studies showed that the p(MMA-co-AM)/PVA@PSO hydrogel had good biocompatibility and significantly accelerated diabetic wound healing by inhibiting excessive MMP-9 in diabetic wounds, increasing growth factor secretion, improving vascularization, increasing collagen deposition and promoting re-epithelialization. Therefore, this study provided a reliable therapeutic strategy for diabetic wound healing, some theoretical basis and new insights for the rational design and preparation of wound hydrogel dressings with high porosity, high drug-loading performance and excellent mechanical properties.

## Graphical abstract

**Figure:**
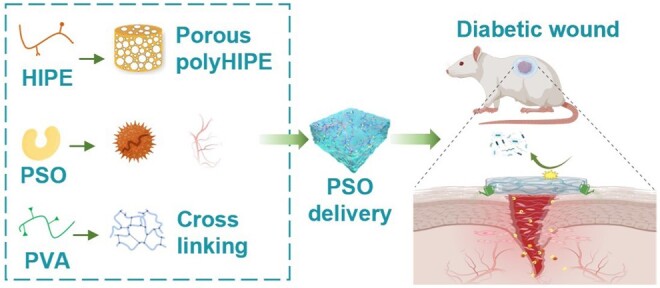

## Introduction

Delayed wound healing is a common and serious complication of diabetes, which often leads to diabetic foot ulcers (DFUs) and seriously affects the physical and mental health of patients [1]. Meanwhile, diabetic ulcers are the most common cause of non-traumatic amputation worldwide and the most expensive type of chronic wound [2, 3]. Although great efforts have been devoted to the treatment of diabetic wounds, it remains a significant challenge to develop efficient approaches to this issue [4]. One of the main factors hindering the healing of diabetic wounds is the overproduction of matrix metalloproteinases (MMPs) in diabetic wounds, caused by high concentration of glucose, aberrant inflammation and oxidative stress [5]. Notably, the amount of MMP-9 is enhanced 14 times more in diabetic ulcers compared to traumatic wounds [6]. The high concentration of MMP-9 further degrades matrix proteins and a variety of growth factors, impairing cells crucial for wound repair, including fibroblasts, endothelial cells and keratinocytes. This results in a reduction of cell proliferation and migration, as well as severe vascular damage, which seriously hinders the healing process of diabetic wounds [6, 7]. Thus, the treatment strategy for diabetic wound healing that focuses on inhibiting excessive MMP-9, reducing extracellular matrix (ECM) and growth factor degradation, reducing cellular and vascular damage, promoting cell proliferation and migration and increasing angiogenesis and collagen deposition is a feasible approach.

Studies have shown that the external drugs currently used for diabetic wound treatment mainly include antibiotics, cell growth factors and chemokines [8]. However, antibiotics only exert antibacterial effects, and the therapeutic effect of local application of antibiotics is not favorable. Moreover, prolonged antibiotic usage escalates bacterial drug resistance and increases the risk of wound infection [9]. Cell growth factors and chemokines are easily inactivated due to changes in external conditions, resulting in a greatly reduced curative effect. Sucrose octasulfate (SOS), as a nano-oligosaccharide factor, exhibits good drug stability and serves multiple roles, including the regulation of MMPs [10], stabilization of related growth factors [11], promotion of angiogenesis [12] and improvement of inflammatory response and oxidative stress [13]. There is strong clinical evidence that the wound dressings added with SOS salt have a good therapeutic effect on diabetic wounds [10, 12]. The related guideline has shown that SOS dressings can improve wound healing in specific types of DFU and recommends them as one of the treatments with evidence-based potential [14]. Moreover, multiple studies have shown that the use of SOS dressings in the treatment of DFU is less costly, more cost-effective and has a higher rate of wound healing [15–17]. However, the concentration of potassium sucrose octasulfate (PSO) in the wound cannot be better controlled by local application alone, and the uneven distribution of the drug may have the opposite therapeutic effect. Thus, using a reliable wound dressing as a carrier to slowly and evenly release PSO and continuously act on the wound is a reasonable way.

Hydrogels are ideal wound dressings that isolate external harmful substances, absorb tissue exudates, create a moist and suitable environment for wounds and transport functional drugs to accelerate wound healing [18, 19]. Especially, the hydrogel with porous networks exhibits a structure similar to the natural ECM, providing a biomimicking moist environment conducive to cell adhesion and growth. It has demonstrated superiority in promoting wound repair [20, 21]. Furthermore, the high porosity and high specific surface area of porous hydrogels are also conducive to drug adsorption and improve drug-loading performance [22–24]. Numerous porous hydrogel materials have been reported, such as metal–organic framework hydrogels [25], porous silica hydrogels [26] and hydrogel microspheres [27]. However, the majority of these hydrogels exhibit relatively low porosity. In contrast, porous hydrogels prepared through the high internal phase emulsion (HIPE) technique often possess interconnected porous structures with high porosity and high specific surface area [28]. Hydrogels are mainly classified into natural product hydrogels and synthetic product hydrogels according to their raw material sources. Most natural hydrogels, such as fibrous protein hydrogels [29], hyaluronic acid hydrogels [30], methylcellulose hydrogels [31] and chitosan hydrogels [32], typically encounter issues, including insufficient mechanical performance, poor stability, low porosity, or complex production process [33, 34]. A good mechanical property is one of the important prerequisites for ensuring the normal function of hydrogels. Most synthetic polymer hydrogels usually exhibit excellent mechanical properties and are easy to adjust in pore structure, drug-loading performance, mechanical properties and chemical properties [35]. Therefore, we decided to design and manufacture a synthetic polymer hydrogel with good mechanical properties and high porosity as a carrier for PSO delivery.

**Scheme 1.**
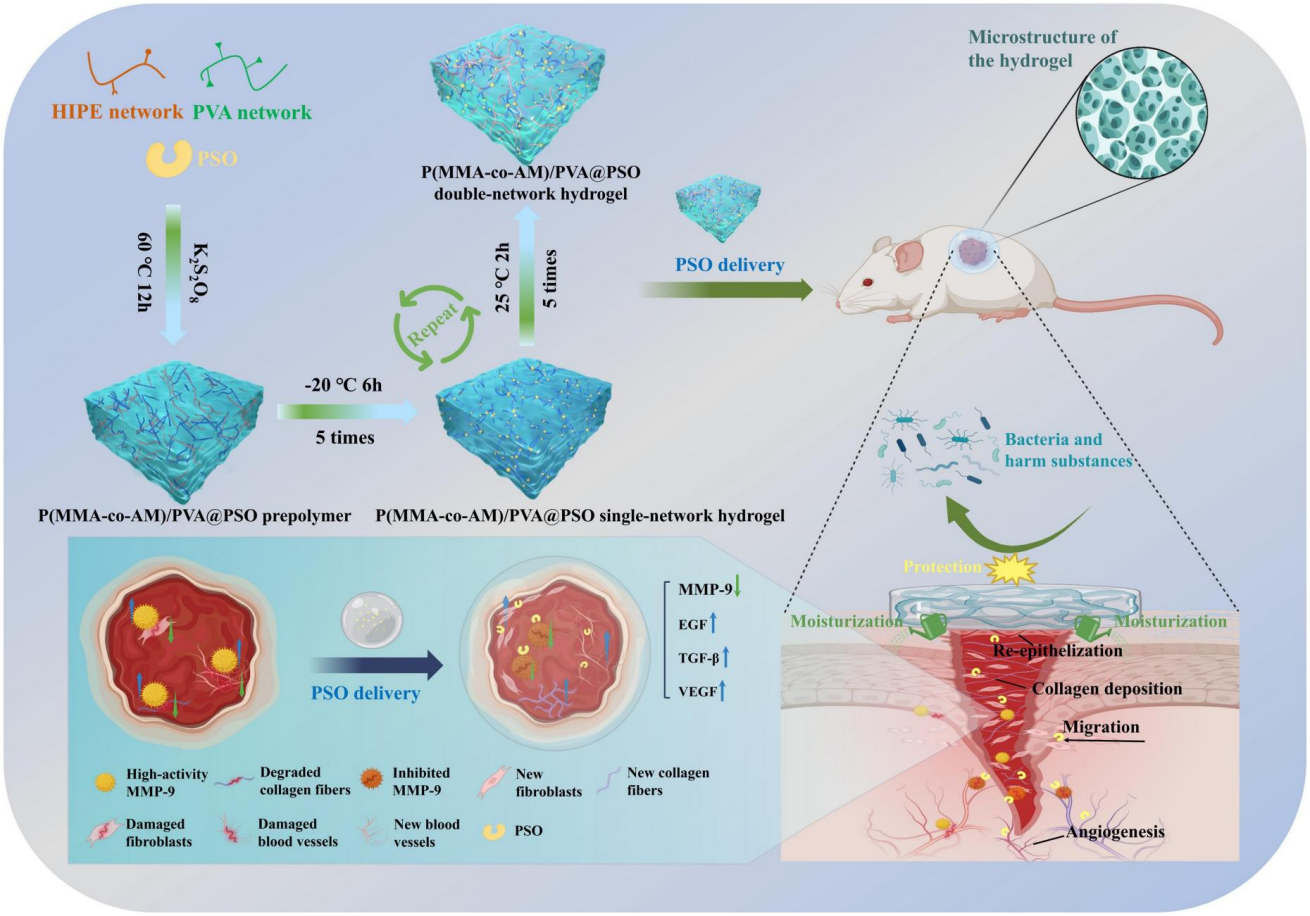
The design strategy of the p(MMA-co-AM)/PVA@PSO hydrogel for treating diabetic wounds.

In this study, we designed and prepared a new double-network porous hydrogel by HIPEs, and employed it as a vehicle for PSO delivery to accelerate diabetic wound healing (Scheme 1). This hydrogel is composed of p(MMA-co-AM) and PVA networks, exhibiting an interconnected pore structure, with good mechanical properties, high porosity and high drug-loading performance. Firstly, the porous p(MMA-co-AM) skeleton structure was synthesized through the HIPE technique to increase the porosity and functional sites of the hydrogel, which facilitates PSO loading and water absorption. Then the PVA network was introduced by cyclic freezing and thawing, allowing PVA to form dynamic hydrogen bonds with the p(MMA-co-AM) network to enhance the mechanical properties. To validate these, the internal structure of the hydrogel was characterized. The mechanical properties of the hydrogel were analyzed by compression and tensile testing. Next, we use the ion chromatograph (ICP) to verify the drug release effect of the hydrogel. Subsequently, the biocompatibility, the promotion of angiogenesis and the promotion of cell proliferation and migration of the hydrogel were validated through cell experiments. Finally, the feasibility of the p(MMA-co-AM)/PVA@PSO hydrogel as a diabetic wound dressing was verified in a diabetic rat wound model, and the potential mechanisms were further analyzed by histological evaluation, immunofluorescence staining and real time quantitative polymerase chain reaction (RT-qPCR) technology. Therefore, this study provided a reliable treatment strategy and some new insights into promoting the early healing of diabetic wounds.

## Materials and methods

### Materials

Acrylamide (AM) was supplied by Macklin (Shanghai, China). Potassium persulfate, trichloromethane and isopropyl alcohol were purchased from Sinopharm Chemical Reagent (Shanghai, China). Potassium sucrose octasulfate was purchased from Avito (Shanghai, China). Polyvinyl alcohol (PVA), methyl methacrylate (MMA), Span-80, divinylbenzene (DVB) and all other chemicals were purchased from Aladdin (Shanghai, China). Cell counting kit 8 was purchased from Biosharp (Wuhan, China). Live/dead staining kits, sodium citrate buffer and streptozotocin were purchased from Solarbio (Beijing, China). The activated partial thromboplastin time assay kit and prothrombin time assay kit were purchased from Rayto (Shenzhen, China). The 5-ethynyl-2’-deoxyuridine (EdU) kit was provided by Beyotime (Shanghai, China). The MMP-9 protein reagent for the *in vitro* cell assay was purchased from Novoprotein (Suzhou, China). The transforming growth factor-β (TGF-β) antibody was provided by Boster (Wuhan, China). The vascular endothelial growth factor (VEGF) antibody and epidermal growth factor (EGF) antibody were purchased from ABclonal (Wuhan, China). The MMP-9 antibody was purchased from Proteintech (Wuhan, China). The platelet endothelial cell adhesion molecule-1 (CD31) antibody was provided by Abcam (Shanghai, China).

### Preparation and characterization of hydrogels

#### Preparation of hydrogels

PVA (0.8 g) was dissolved in deionized water with stirring at 80°C. MMA, AM, DVB, potassium persulfate, Span80 and deionized water were added into the centrifuge tube according to the proportions in Table 1. Then stir well and place in the oven at 60°C for the first step of polymerization into HIPE. The prepolymer was frozen at −20°C for 6 hours and thawed at 25°C for 2 h. The p(MMA-co-AM)/PVA hydrogel can be prepared by repeated freezing and thawing five times. Px refers to the proportion of PVA in the double-network hydrogel, and Hx refers to the proportion of the dispersed phase in HIPE. The preparation of p(MMA-co-AM)/PVA@PSO hydrogel with different PSO concentrations is added to the PSO (0.1–0.5 g) in the first phase of the above synthesis process, and the rest of the steps are the same.

**Table 1.** Composition parameters for the prepared HIPEs

Sample	Continuous phase	Dispersed phase	PVA (g)
	AM (g)/MMA (ml)/DVB (ml)/Span 80 (ml)/K_2_S_2_O_8_ (g)	Water (ml)	
P8-H80	0.47/0.65/0.85/0.1/0.1	8.0	0.8
P8-H85	0.36/0.50/0.64/0.1/0.1	8.5	0.8
P8-H90	0.24/0.30/0.42/0.1/0.1	9.0	0.8
P8-H95	0.12/0.17/0.21/0.1/0.1	9.5	0.8

#### Structural characterization of hydrogels

A Fourier transform infrared (FTIR) spectrometer (IS5, Thermo, USA) was used to scan in the range of 4000–500cm^−1^ to characterize p(MMA-co-AM)/PVA, p(MMA-co-AM)/PVA@PSO and PSO. The microscopic morphology of the hydrogels was observed using scanning electron microscopy (SEM) (JSM-IT200, JEOL, Japan). Briefly, the hydrogel was immersed in PBS to reach swelling equilibrium, then placed in a freeze dryer for freeze-drying. The freeze-dried hydrogel sample was sputter-coated with gold and observed under SEM.

#### PSO release detection from p(MMA-co-AM)/PVA@PSO hydrogel

The p(MMA-co-AM)/PVA@PSO hydrogel (1 g) was cut and placed in PBS, deionized water, and 75% alcohol. The released liquids were collected for detection. Detection was performed using an ion chromatograph (ICS600, Thermo Fisher). On average, three replicated tests were used to plot the release results.

### *In vitro* experiments

#### Cell biocompatibility evaluation

The extracts of hydrogels were used to evaluate the hydrogels’ biocompatibility. The hydrogel was initially immersed in 75% medical alcohol for 1 h, followed by a subsequent soaking in deionized water for an additional hour. The deionized water was replaced three times to eliminate any residual alcohol. After sterilizing, the hydrogels were soaked in the complete cell culture medium at a concentration of 0.1 g/ml for 48 h. NIH3T3 cells were incubated for 24 h and then co-cultured with the extracts of different hydrogels for 24 and 48 h, respectively. The cell viability of NIH3T3 cells in different treatments was detected by the cell counting kit 8 (CCK 8), and each test was repeated six times. The cytotoxicity of hydrogels was verified by living and dead cell staining. In short, the NIH3T3 cells were co-cultured with hydrogel extracts for 24 and 72 h, then stained with a live/dead staining kit and observed under the fluorescence microscope (Soptop ICX41, Sunny Optical Technology Co., Ltd).

#### Hemolytic and coagulation activity testing of hydrogels

Red blood cells were isolated from fresh blood collected by rats by centrifugation (1000 rpm) for 5 min and then diluted to 5% with a PBS solution (pH = 7.4). Hydrogel was mixed with the prepared red blood cell solution and placed at a constant temperature of 37°C for 1 h, then the treated mixture was centrifuged (1000 rpm, 5 min). The absorbance of the supernatant was measured at 540 nm by an enzyme labeling instrument (FC, Thermo Fisher Scientific). The cells treated with PBS solution were used as a negative control, and the cells treated with ultra-pure aqueous solution were used as a positive control. According to the methods in the literature [36], the hydrogel extract (0.1 μg/ml, PBS pH7.4, triplicates) was incubated with plasma. The prothrombin time (PT) and activated partial thromboplastin time (APTT) were determined using an automated coagulation analyzer (Chemray, China).

### *In vivo* experiments

#### The diabetic rat model of full skin defects

The animal experiments in this study followed the guidelines set for experimental animal management and welfare ethics at the Zhongnan Hospital of Wuhan University (China). The ethical approval number was ZN2022198. The rat model of type 2 diabetes was induced by an intraperitoneal injection of streptozotocin (STZ) combined with a high-fat and high-sugar diet [37]. Blood glucose was measured on the 3rd and 7th days. If the blood glucose was stable above 16.6 mmol/l, the diabetes modeling was considered successful. The blood glucose of SD rats was recorded until the wound healed completely. Diabetic rats were randomly divided into four groups (*n* = 6). After the rats were anesthetized and shaved, four full skin defect wounds (10 mm in diameter) were formed on the back of each rat. After rinsing with normal saline, the rats were treated in different ways: (1) dressing the wounds with gauzes (the gauze group/the control group), (2) treating the wounds with the 3 m Tegaderm hydrogel dressing (the 3 m hydrogel group), (3) treating wounds with the p(MMA-co-AM)/PVA hydrogel (the hydrogel I group) and (4) treating wounds with the p(MMA-co-AM)/PVA@PSO hydrogel (the hydrogel II group). The hydrogels and gauzes were changed every 48 h during the healing process.

#### Histological and immunofluorescence analysis

Skin tissues were collected on Days 7 and 14 and fixed overnight with 4% paraformaldehyde and embedded in paraffin. After sectioning, samples were stained with hematoxylin–eosin (HE) and Masson's trichrome solution (MTS). Immunofluorescent staining was performed using regenerated wound skin collected on Day 7. The sections were stained with CD31, MMP-9, EGF, TGF-β and VEGF antibodies, respectively. All slides were observed under a microscope (IX53, Olympus, Japan) and analyzed under standard procedures.

### Statistical analysis

All data were expressed as mean ± standard deviation (SD), and the statistical significance was calculated using GraphPad Prism 8.0 (Graph-Pad Software, CA, USA). A Student's *t*-test, or analysis of variance (ANOVA), was selected to determine statistical significance. Differences with *P* values of less than 0.05 indicated significance. The results of the significant difference were: n.s.: no statistical difference, *: *P* < 0.05, **: *P* < 0.01, ***: *P* < 0.005, ****: *P* < 0.001.

## Results and discussion

### Design and preparation of hydrogels

**Figure 1.**
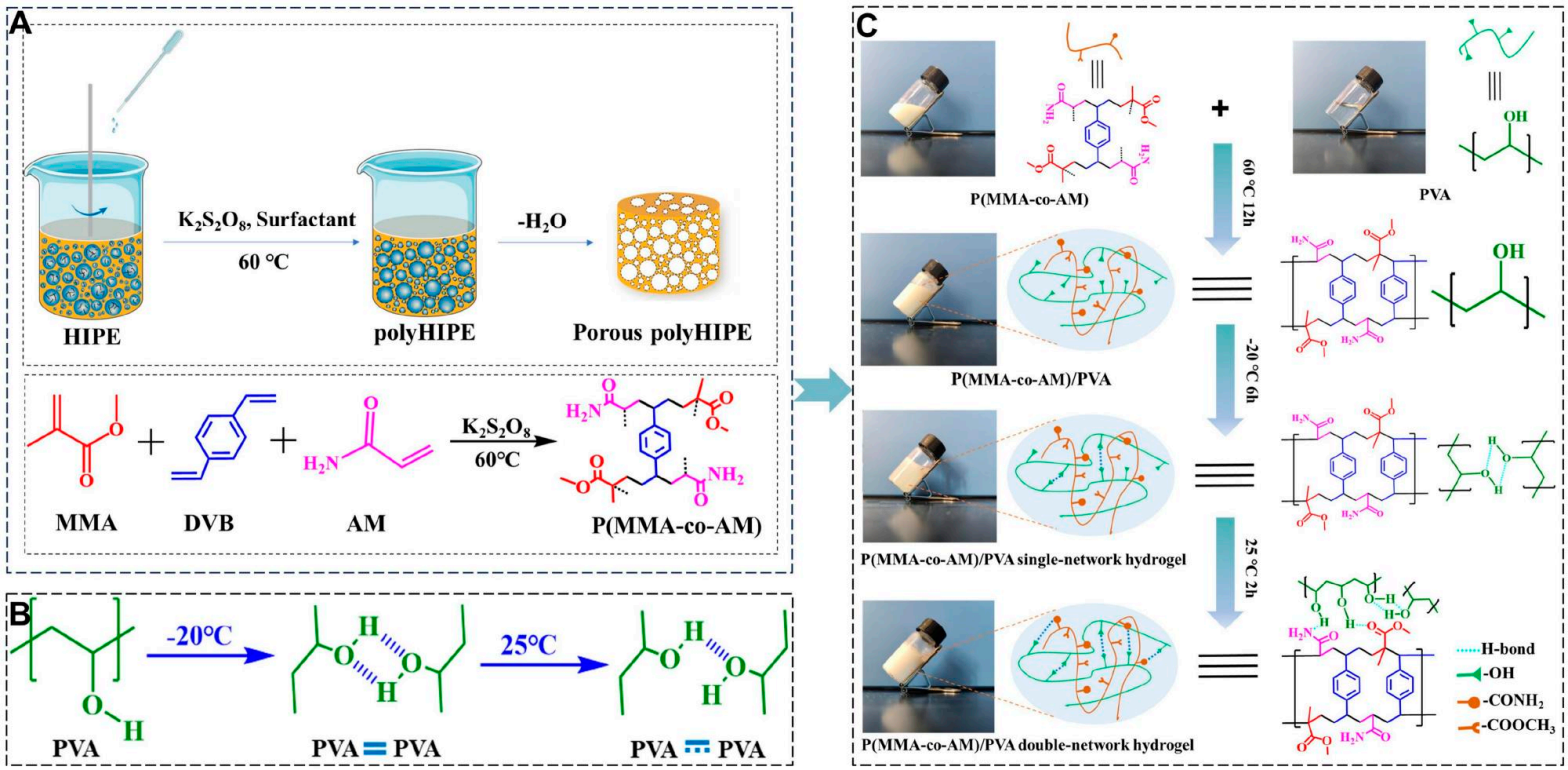
Preparation of the p(MMA-co-AM)/PVA double-network hydrogel. (**A**) Construction of the porous skeleton structure through HIPE. (**B**) Cross-linking process of the PVA network. (**C**) Detailed process for preparing the p(MMA-co-AM)/PVA hydrogel.

The HIPE technique is a good method for preparing porous polymer materials and has been applied in the synthesis of various porous polymers. In the biomedical field, polyHIPE materials have shown great potential in drug delivery, bioconjugation, cell culture and tissue scaffolds [38–40]. In this study, we prepared a HIPE-based double-network porous hydrogel composed of p(MMA-co-AM) and PVA through a continuous reaction method (Figure 1). MMA, DVB, AM, Span80, potassium persulfate and deionized water constitute a HIPE system. Among them, Span80 is adsorbed on the phase interface as a surface-active substance to reduce the interface free energy and form an interface film with high mechanical strength to achieve the dynamic stability of the system. Next, under the catalysis of potassium persulfate, MMA, DVB and AM were facilitated to undergo free radical polymerization to synthesize p(MMA-co-AM) with an interconnected porous structure (Figure 1A). Among them, p(MMA-co-AM) plays a crucial role in forming a skeleton structure of connected pores. This structure not only enhances the hydrogel's porosity and specific surface area but also increases the number of functional sites within the hydrogel. These enhancements prove advantageous for drug adsorption and water absorption performance. Then, the PVA network was introduced through the cyclic freezing-thawing process (Figure 1B). PVA possesses the property of rapid gelation after repeated freezing and thawing [41]. Consequently, during the iterative freezing and thawing steps, PVA and p(MMA-co-AM) undergo cross-linking (Figure 1C), thereby enhancing the hydrogel’s cross-linking degree and subsequently improving its mechanical properties. This ensures the hydrogel’s reliability in practical applications, safeguarding it against deformation or breakage from external forces. The introduction of PSO serves to inhibit MMPs and promote angiogenesis. After the above treatment process, the p(MMA-co-AM)/PVA hydrogel with both polyHIPE features (high porosity, high specific surface area and multifunctional groups) and the high flexibility of PVA can be prepared.

### Structural characterization and swelling properties of hydrogels

**Figure 2.**
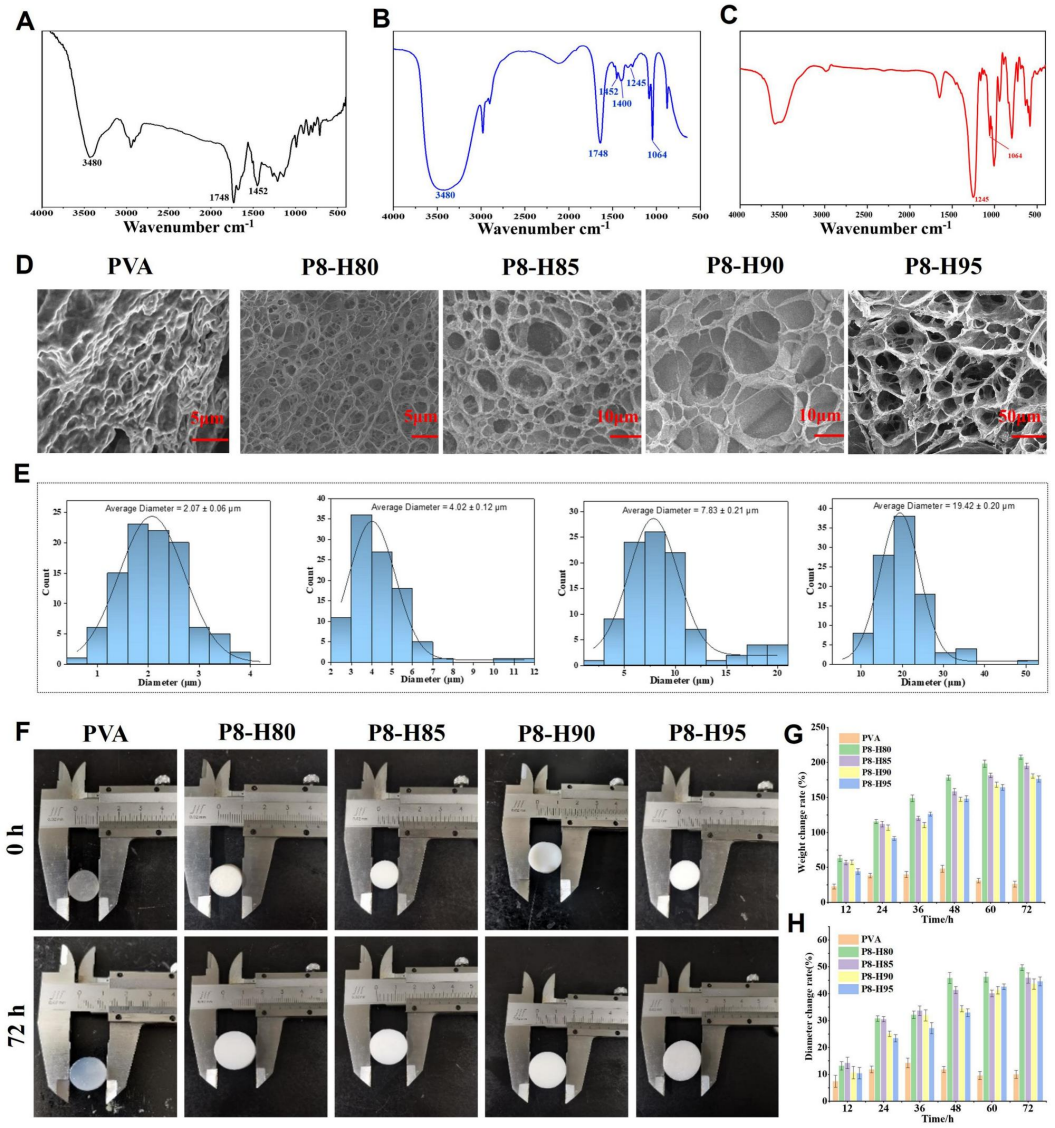
The FITR spectra, SEM images and swelling properties of different hydrogels. (**A–C**) The FITR spectra of p(MMA-co-AM)/PVA, p(MMA-co-AM)/PVA@PSO, PSO. (**D**) The SEM images of different hydrogels. (**E**) Quantitative analysis of pore sizes of P8-H80, P8-H85, P8-H90 and P8-H95 double-network hydrogels. (**F**) The morphological change of different hydrogels before and after swelling in PBS for 72 h. (**G**) The weight and (**H**) diameter change rate of different hydrogels before and after swelling in PBS for 12, 24, 36, 48, 60 and 72 h.

FTIR was used to characterize the molecular structures of p(MMA-co-AM)/PVA, p(MMA-co-AM)/PVA@PSO and PSO. Figure 2A and B shows that both p(MMA-co-AM)/PVA and p(MMA-co-AM)/PVA@PSO demonstrated a benzene ring skeleton vibration peak at 1748 cm^−1^, a C–H stretching vibration peak of the benzene ring at 1452 cm^−1^ and a N–H stretching vibration at 3480 cm^−1^, indicating that p(MMA-co-AM)/PVA was successfully synthesized. Moreover, the ${\text{SO}}_{4}^{2-}$ peak (1245 cm^−1^) and –COOR peak (1064 cm^−1^) were visible in both PSO and the p(MMA-co-AM)/PVA@PSO hydrogel, which indicated that PSO has been successfully loaded into the p(MMA-co-AM)/PVA hydrogel (Figure 2B and C). The thermogravimetric analysis was carried out to detect the thermal stability of the hydrogels. From the TG-DTG curve of the sample (Supplementary Figure S1), it can be seen that the initial thermal decomposition temperature of the PVA hydrogel was only 254.15°C, the initial decomposition temperature of p(MMA-co-AM) was 361°C, while the initial decomposition temperature of p(MMA-co-AM)/PVA reached 374.88°C, indicating the better thermal stability of the double-network hydrogel. The DSC-TG curve of the P8-H95 hydrogel further illustrated a first small step between 45 and 190°C with a mass loss rate of 6.214%, which was exactly the mass loss of p(MMA-co-AM) in the double-network hydrogel. After 167°C, a second step appeared with a loss rate of 83.46%, representing the decomposition loss of PVA. Additionally, the DSC curve exhibited two decomposition peaks at 191.45°C and 428.46°C, which is exactly consistent with the decomposition temperature of the TG curve. This result confirmed the successful preparation of the double-network hydrogel.

The interconnected porous structure can increase the porosity and specific surface area of the hydrogel, which has great advantages for drug loading and improving water absorption performance [22]. At the same time, a suitable porous structure can also simulate the ECM, which helps cell adhesion and growth [42]. The microstructure of the hydrogel was observed by SEM. As shown in Supplementary Figure S2, p(MMA-co-AM) exhibited a porous morphology with dense and interconnected pores, which was a typical microstructure of polyHIPE. In addition, all the p(MMA-co-AM)/PVA double-network hydrogels exhibited interconnected pore structures, and the pores were denser and more uniform, while the PVA single-network hydrogel does not have such a pore structure (Figure 2D). Interestingly, the pore size and pore density of the double-network hydrogel can be adjusted by adjusting the proportion of the dispersed phase in HIPE. As the dispersed phase increased, the pore size of the hydrogel gradually increased (Figure 2E). This is mainly because as the proportion of the dispersed phase increases, the emulsion particles formed by the continuous phase become larger, and the cavity formed after the continuous phase curing will also become correspondingly larger. Therefore, according to this interesting characteristic, the pores can be adjusted according to the actual needs of the production process to increase the applicability and functionality of the hydrogel.

The equilibrium swelling ratio reflects the internal cross-linking degree and the structural characteristics of the hydrogels [43]. The morphological changes of different hydrogels after 72-hour swelling are shown in Figure 2F. There were only minor changes in the weight and diameter of the PVA single-network hydrogel, and the weight and diameter after soaking for 48 h decreased compared with those before 48 h (Figure 2G and H). This is mainly attributed to the fact that water initially permeated the hydrogel network to expand its volume during the swelling process. The cross-linking degree of PVA single-network hydrogel is not as high as that of double-network hydrogel, making its ability to resist volume expansion weak and leading to internal bursting [33]. However, the weight and diameter of all double-network hydrogels gradually increased during the 72-h soaking process without any decrease, and the weight change rate of the double-network hydrogels reached 200%, while the maximal change rate of diameter was only about 50% (Figure 2G and H), indicating stronger water absorption properties. This is attributed to the fact that the porous structure constructed through the HIPE increases the porosity of the hydrogel [33]. Additionally, to evaluate the influence of pH environments in both normal skin and injured skin on the swelling properties of the hydrogels, we evaluated the swelling properties of hydrogels at pH 5 and pH 8, respectively. The results revealed that the hydrogel’s swelling capacity showed no significant changes in pH 5 and pH 8 environments compared to pH 7 (Supplementary Figure S4).

### Mechanical characterization of hydrogels

**Figure 3.**
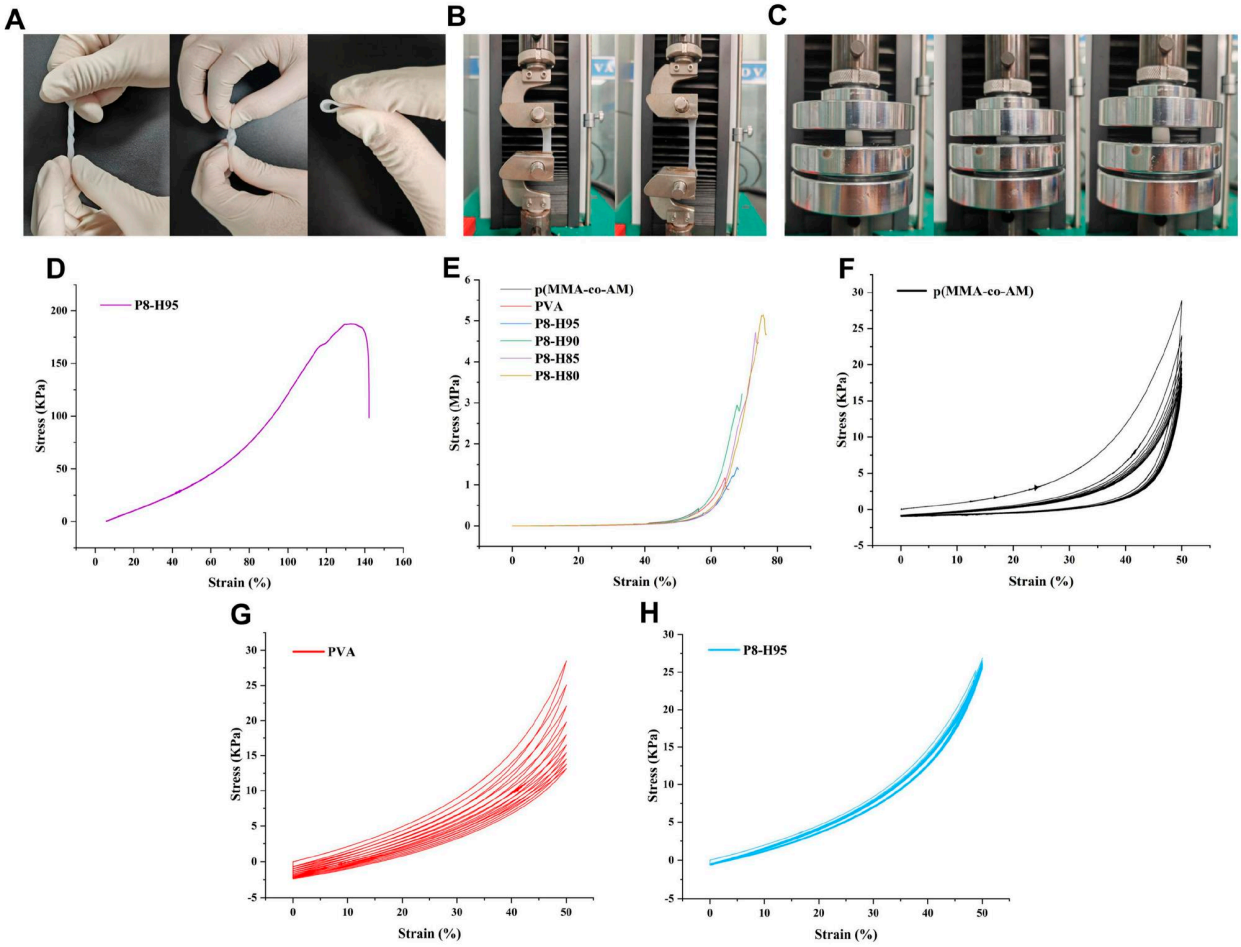
Mechanical characterizations of different hydrogels. (**A**) The complex mechanical properties of p(MMA-co-AM)/PVA (P8-H95) hydrogel under different deformation conditions: knotting, twisting and folding. (**B**) Process of the tension performance testing. (**C**) Process of the compression performance testing. (**D**) The tension stress–strain curve of p(MMA-co-AM)/PVA (P8-H95) hydrogel at strains from 0% to fracture. (**E**) The compression stress–strain curves of different hydrogels. (**F–H**) Loading–unloading cyclic compressive stress–strain curves of the p(MMA-co-AM), PVA (P8), and p(MMA-co-AM)/PVA (P8-H95).

Good mechanical properties are one of the important prerequisites for hydrogel wound dressings to exert their therapeutic effects [44]. Meanwhile, an ideal hydrogel dressing must have both strong mechanical properties and appropriate softness. Therefore, we selected the softer P8-H95 hydrogel as a representative p(MMA-co-AM)/PVA hydrogel for mechanical property testing. Narrow hydrogel samples were prepared to simply display their complex mechanical properties, such as bending and twisting, under different deformation conditions. It can be seen that the P8-H95 hydrogel can be twisted, knotted, or folded (Figure 3A). Tensile testing further investigated the tensile properties of the P8-H95 hydrogel (Figure 3B). As shown in Figure 3D, the tensile strain of the P8-H95 hydrogel can reach 145%, and the maximum tensile stress is about 120 KPa. These results indicated that the P8-H95 hydrogel had good flexibility and toughness.

Compressive tests were conducted to evaluate the compressive capacity of p(MMA-co-AM), PVA (P8) and p(MMA-co-AM)/PVA hydrogels (Figure 3C). The compressive stress–strain of different hydrogels is shown in Figure 3E. Specifically, the p(MMA-co-AM) single-network hydrogel fragmented when the strain reached 53%, and the maximum compressive strength was 0.43 MPa. The maximum compressive strength of PVA single-network hydrogel was 1.17 MPa, and the maximum strain was 64.2%. All double-network hydrogels show stronger compression properties compared with other two single-network hydrogels. The maximum compressive strengths of P8-H95, P8-H90, P8-H85 and P8-H80 hydrogels were 1.48, 2.82, 4.60 and 5.13 MPa, and the maximum strains were 68.2%, 69.4%, 73.4% and 76.3%, respectively.

Next, we conducted loading-unloading tests to study the fatigue resistance of different hydrogels. In Figure 3F, the p(MMA-co-AM) single-network hydrogel showed a very obvious hysteresis loop during the first loading-unloading process under 50% compressive strain, and its shape changed significantly. The stress–strain curve of PVA hydrogel's cyclic compression also showed obvious separation (Figure 3G). However, the stress–strain curve of the P8-H95 hydrogel overlapped well (Figure 3H), indicating the double-network hydrogel possessed strong fatigue resistance. In summary, the results showed that p(MMA-co-AM)/PVA double-network hydrogel has good compressive properties, tensile properties and fatigue resistance.

### Release of PSO from the p(MMA-co-AM)/PVA@PSO hydrogel *in vitro*

The P8-H95 hydrogel had a suitable softness, excellent mechanical properties, good water absorption properties, and bigger, dense and uniform pores. As a result, the P8-H95 hydrogel was selected as the drug-binding carrier. Moreover, cell experiments demonstrated that the P8-H95 hydrogel with a drug content of 4% w/v contained a greater amount of drug and had good biocompatibility (Supplementary Figure S5). Therefore, the p(MMA-co-AM)/PVA@PSO hydrogel (95% HIPE, 8% PVA and 4% w/v PSO) was selected for further drug release detection and biological applications.

**Figure 4.**
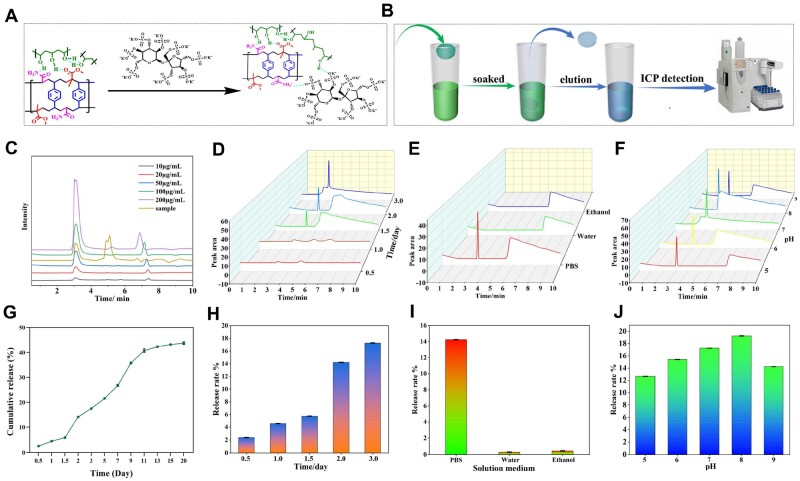
PSO Loading and release of the p(MMA-co-AM)/PVA@PSO hydrogel. (**A**) The principle of combining PSO with the p(MMA-co-AM)/PVA hydrogel. (**B**) The process of PSO release detection. (**C**) The standard ICP spectrum of PSO. (**D**) Detection of PSO release at different time points. (**E**) Detection of PSO release in different solution mediums. (**F**) Detection of PSO release in different pH environments. (**G**) The PSO cumulative release of the p(MMA-co-AM)/PVA@PSO hydrogel for 20 days. (**H**) Quantitative analysis of PSO cumulative release at different time points. (**I**) Quantitative analysis of PSO release in different solution mediums. (**J**) Quantitative analysis of PSO release in different pH environments.

The ICP was used to investigate PSO release. The principle of drug adsorption and the detection steps are shown in Figure 4A and B, respectively. The linear equation for quantitative detection of PSO release is shown in Supplementary Figure S6. The peak of the standard solution appeared at 3.1 min, and the sample solutions also had an obvious peak at this retention time point (Figure 4C), indicating the sample solutions contained the target drug and further demonstrating that PSO was successfully loaded into the p(MMA-co-AM)/PVA hydrogel. The results of the cumulative PSO release at different time points are shown in Figure 4D and H. To explore the long-term PSO release effect of the hydrogel, we extended the detection time to 20 days (Figure 4G). As the soaking time increased, the release amount of PSO increased gradually, which indicated the p(MMA-co-AM)/PVA@PSO hydrogel could sustainably release PSO.

In addition, we investigated the hydrogel’s drug release in different liquids. The release of PSO was very little in deionized water and 75% ethanol, while it was more in PBS solution (Figure 4E and I). This is probably due to the effect of salt ions on drug release. ${\text{HPO}}_{4}^{2+}$, H_2_PO^4−^, Cl^−^ and Na^+^, etc., in PBS increased the total ion concentration of the solution, which reduced the probability of PSO binding to the material and increased the dissociation degree of PSO in the solution, thereby achieving the goal of drug release. A large number of ions in diabetic wound exudate guarantees the effective release of PSO.

Generally, the exudates of a normal acute wound are weakly acidic (pH = 4–6), while the exudates of a chronic wound are weakly alkaline (pH = 7–9) [45]. Therefore, it is necessary to investigate the effects of different pH environments on PSO release. The hydrogel release fluid samples were collected on 3rd day for testing. As depicted in Figure 4F and J, as pH increased, the drug release peaked at a pH of 8, decreased gradually and maintained a good effect of drug release. Therefore, the p(MMA-co-AM)/PVA@PSO hydrogel had a good PSO release property in weakly acidic, neutral and weakly alkaline environments, as well as a good adaptability to wound pH changes.

### Biocompatibility and tube formation of p(MMA-co-AM)/PVA@PSO hydrogel *in vitro*

**Figure 5.**
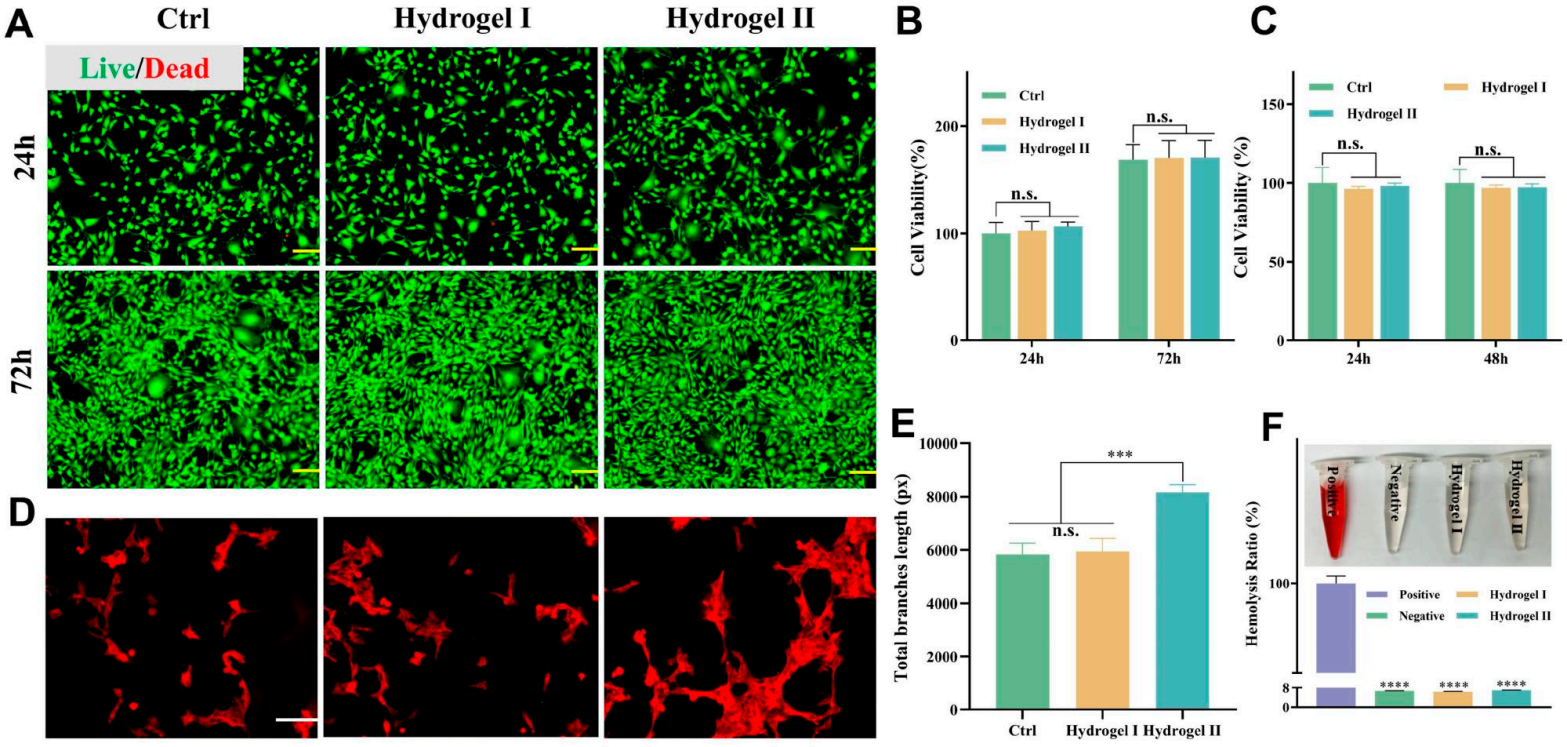
The biocompatibility and angiogenesis ability of hydrogels *in vitro*. (**A**) Live/dead staining of NIH3T3 cells cultured with different hydrogels in a normal environment for 24 h and 72 h, scale bar: 100 μm. (**B**) Cell viability of NIH3T3 cells cultured with different hydrogels in a normal environment for 24 and 72 h. (**C**) CCK-8 assay statistical graph of the effects of different hydrogels on NIH3T3 cell viability for 24 and 48 h. (**D**) The effect of hydrogels on HUVECs tube formation was evaluated by tubule formation assay, scale bar: 100 μm. (**E**) Summarized data of the total length of HUVECs tubule branches. (**F**) The photo and quantitative analysis of the hemolytic activity of different hydrogels. Note: Hydrogel I: the p(MMA-co-AM)/PVA hydrogel; hydrogel II: the p(MMA-co-AM)/PVA@PSO hydrogel. Data are shown as mean ± SD (*n* = 3), *P < 0.05, **P < 0.01, ***P < 0.005, ****P < 0.001, n.s.: no significance.

Biocompatibility and cytotoxicity are crucial considerations in biological materials [46]. CCK-8 and live/dead cell staining analyses were conducted to investigate biocompatibility and cytotoxicity using NIH3T3 cells. Live/dead cell staining showed that both p(MMA-co-AM)/PVA hydrogel (hydrogel I) and p(MMA-co-AM)/PVA@PSO hydrogel (hydrogel II) were highly biocompatible (Figure 5A and B). In addition, the results of the CCK-8 assay showed no significant difference in cell viability between the hydrogels I and II groups and the control group (Figure 5C).

Good biocompatibility requires not only no harm to local tissues and cells but also no harm to blood. A hemolysis test was performed to investigate the hemocompatibility of the hydrogels [47]. Figure 5F exhibits that all of the hydrogel groups’ solutions were observed to be colorless and transparent, while the ultrapure water group showed a bright red color. The hemolysis ratios of the hydrogels I and II exhibited no obvious increase (6.36 ± 0.102% and 6.98 ± 0.07%) compared to the PBS solution (6.72 ± 0.066%). In addition, we further analyzed the effect of the hydrogel on the coagulation *in vitro*. As shown in Supplementary Figure S7, the results of PT and APTT in the hydrogel-treated groups did not exhibit significant differences compared to those in the control group (*P* > 0.05). These results indicate that the hydrogels had good hemocompatibility. Therefore, the p(MMA-co-AM)/PVA@PSO hydrogel has good safety and great potential as wound dressings.

The tubule formation test was used to evaluate the effect of hydrogel II on the angiogenesis of HUVECs. According to the experimental results, it was observed that the hydrogel II group significantly promoted the angiogenesis of HUVEC compared with the control group and the hydrogel I group (Figure 5D). After incubation for 24 h, many different tubules were formed in the hydrogel II group, but rarely in the control group and hydrogel I group (Figure 5E).

### p(MMA-co-AM)/PVA@PSO hydrogel promoted cell proliferation and migration in a simulated diabetic wound environment

**Figure 6.**
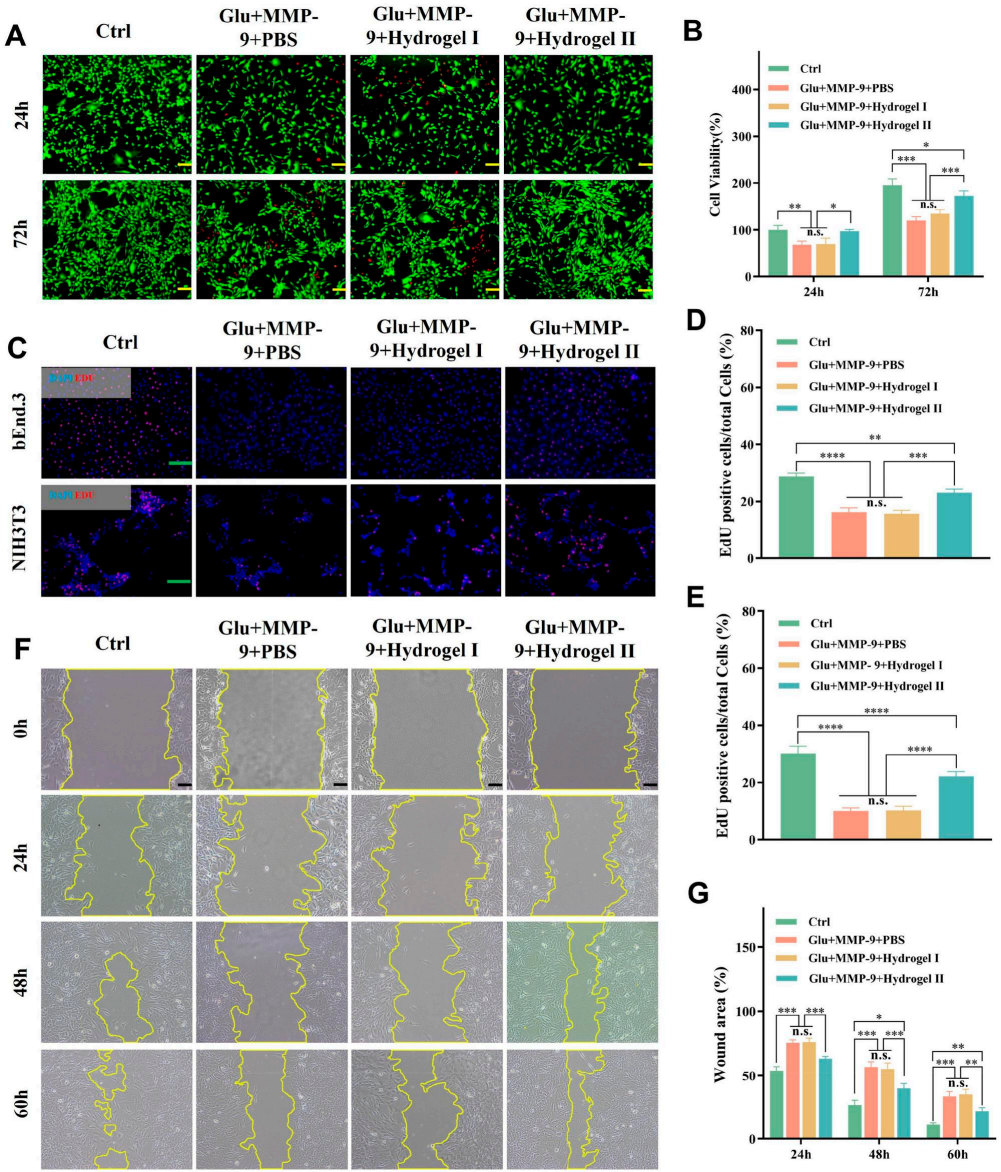
The cell proliferation and migration of hydrogels in a simulated diabetic wound environment. (**A**) Live/dead staining of NIH3T3 cells cultured with different hydrogels in a simulated diabetic wound environment for 24 and 72 h, scale bar: 100 μm. (**B**) Cell viability of NIH3T3 cells cultured with different hydrogels in a simulated diabetic wound environment for 24 and 72 h. (**C**) EdU staining of bEnd.3 and NIH3T3 cells, scale bar: 200 μm. (**D, E**) Relative amount of EdU-positive bEnd.3 and NIH3T3 cells. (**F**) Cell migration of NIH3T3 cells cultured in a simulated diabetic wound environment for 24, 48 and 60 h, scale bar: 100 μm. (**G**) Quantification of NIH3T3 cell migration for 24, 48 and 60 h. Note: Hydrogel I: the p(MMA-co-AM)/PVA hydrogel; hydrogel II: the p(MMA-co-AM)/PVA@PSO hydrogel. Data are shown as mean ± SD (*n* = 3), *P < 0.05, **P < 0.01, ***P < 0.005, ****P < 0.001, n.s.: no significance.

High levels of MMP-9 in diabetic wounds increase the degradation of the ECM and various growth factors, which directly or indirectly affect cell growth [5, 6, 48]. Therefore, we simulated the diabetic wound environment *in vitro* and used the NIH3T3 cells to evaluate the effects of the environment and the hydrogel II on the cells. As shown in Figure 6A, many living cells were seen, and almost no dead cells in the control group. However, there were a large number of dead cells in the Glu + MMP-9 + PBS group and the Glu + MMP-9 + Hydrogel I group, and living cell density was lower. There were a few dead cells in the Glu + MMP-9 + Hydrogel II group, but compared with the control group, the density of living cells decreased (Figure 6B). The EdU cell proliferation kit was used to treat fibroblasts (NIH3T3) and microvascular endothelial cells (bEnd.3) to further evaluate the effect of hydrogel II on cell proliferation. As shown in Figure 6C, a few EdU-positive cells (red fluorescence) were in the Glu + MMP-9 + PBS group and the Glu + MMP-9 + Hydrogel I group, whereas there were many EdU-positive cells in the control group and the Glu + MMP-9 + Hydrogel II group. Quantitative analysis results showed that the cell proliferation activity of the other three groups decreased compared to the control group. Moreover, compared with the Glu + MMP-9 + PBS group and the Glu + MMP-9 + Hydrogel I group, the cell proliferation activity of the Glu + MMP-9 + Hydrogel II group increased (Figure 6D and E). Thus, these results demonstrated that in the simulated environment, high concentrations of glucose and MMP-9 could damage cells and substantially inhibit cell growth, whereas hydrogel II could promote cell proliferation. This could be attributed to the release of PSO by hydrogel II, which inhibited the activity of MMP-9 to reduce the degradation of ECM and growth factors and reduce cell damage [10, 49].

Cell migration is known to play an important role in remodeling the microenvironment in wounds and mediating wound regeneration [50]. The effect of hydrogel II on fibroblast migration was explored by the scratch assay. It can be observed from the photos (Figure 6F) and histograms (Figure 6G) that the remaining areas of cell scratches in the control group were always the smallest at different times, compared with other groups, and the scratch was almost completely healed at 60 h. Compared to the Glu + MMP-9 + PBS group and the Glu + MMP-9 + Hydrogel I group, the remaining areas of scratches in the Glu + MMP-9 + Hydrogel II group were smaller. This could be attributed to the effective release of PSO from hydrogel II [10, 49].

### p(MMA-co-AM)/PVA@PSO hydrogel promoted diabetic wound healing *in vivo*

**Figure 7.**
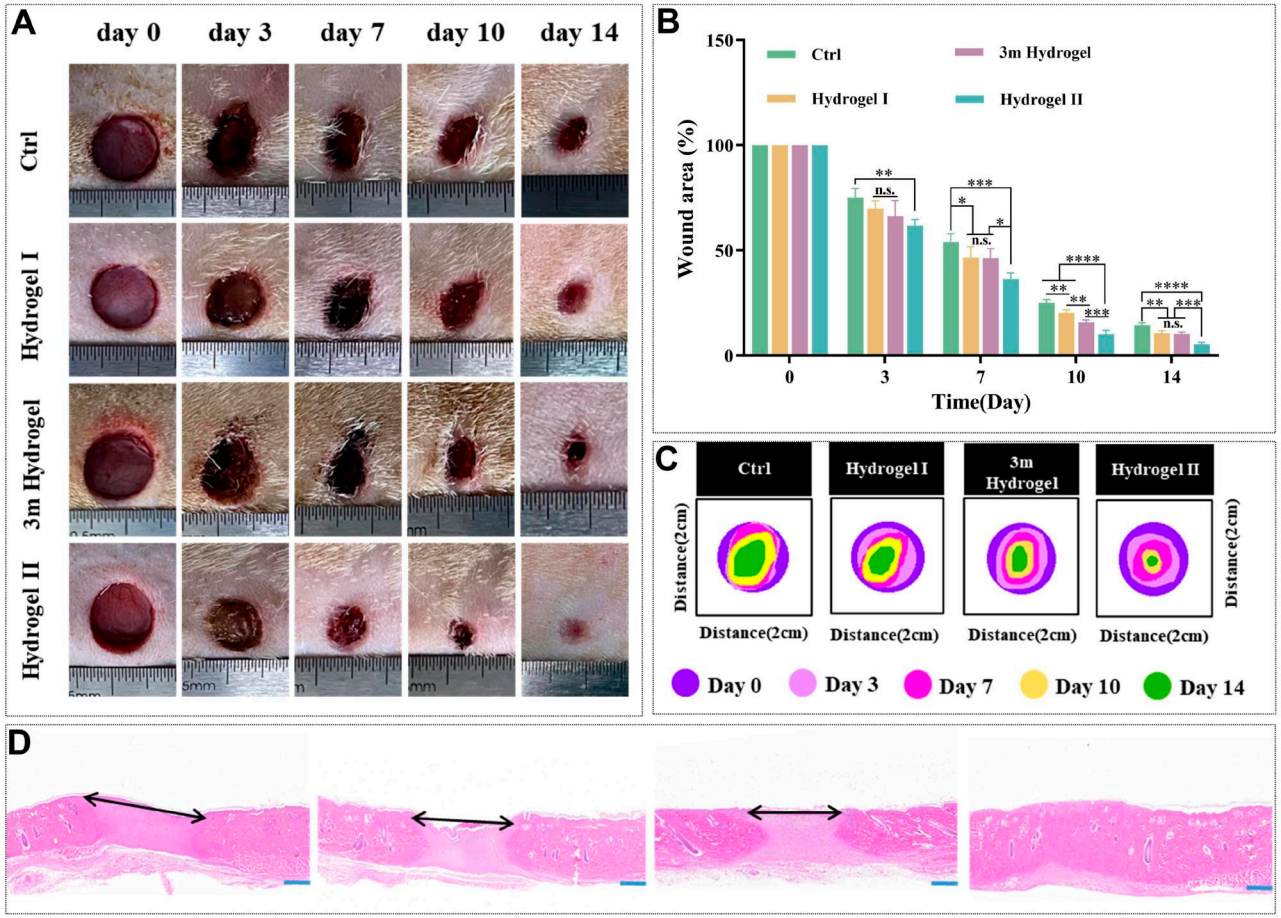
Analysis of skin wound healing in diabetic rats. (**A**) Photos of the wounds with different treatments at Days 0, 3, 7, 10 and 14. (**B**) The quantitative analysis of the remaining wound area at Days 0, 3, 7, 10 and 14. (**C**) Analysis of wound healing trace on Days 0, 3, 7, 10 and 14. (**D**) HE staining of healed skin tissues on Day 14. The black arrow shows the area without healing, scale bar: 1 mm. Note: Hydrogel I: the p(MMA-co-AM)/PVA hydrogel; hydrogel II: the p(MMA-co-AM)/PVA@PSO hydrogel. Data are shown as mean ± SD (*n* = 3), *P < 0.05, **P < 0.01, ***P < 0.005, ****P < 0.001, n.s.: no significance.

Hydrogels are widely used as skin wound dressings to provide a humid environment, isolate external harmful factors and accelerate tissue repair [51]. The ‘3 m’ commercial hydrogel is often used in the treatment of chronic wounds, so we used it as a positive control group [52]. As shown in Figure 7A and C, the remaining wound area of each group decreased over time. On the 3rd day, obvious scars were observed in all groups. Compared with the control (ctrl) group, the wound area was significantly smaller in the hydrogel II group. Nevertheless, there was no significant difference between the 3 m hydrogel, the hydrogel I and the gauze groups (Figure 7B). On the 7th and 10th days, the wound area of each treatment group was further reduced, among which the hydrogel II treatment group had the smallest remaining area and exhibited the highest wound healing rate. Moreover, the wounds treated with the 3 m hydrogel and the hydrogel I were also smaller than that of the ctrl group after the 7th day. This was the reason why the hydrogels maintained the moist environment of the wound and simulated ECM [53]. On the 14th day, it was observed that the wound in the hydrogel II group almost healed completely, and the epithelial cover was the most complete, while the other three groups were still covered with crusts, indicating that the wound was not completely healed. The proportion of residual wound areas on the 14th day was 5.47 ± 0.71%, 14.40 ± 1.14%, 10.40 ± 1.09% and 10.34 ± 0.74% in the hydrogel II, ctrl, hydrogel I and 3 m hydrogel groups, respectively (Figure 7B).

The regenerated skin wounds on the 14th day were collected and stained with hematoxylin-eosin (HE), and the wound healing was further observed under a microscope. The remaining wound area was the smallest in the hydrogel II treatment group, and the wound almost healed completely, while the other three groups showed incomplete wound healing (Figure 7D). The HE results were consistent with the photos taken by digital cameras.

### Histological evaluation of wound regeneration

**Figure 8.**
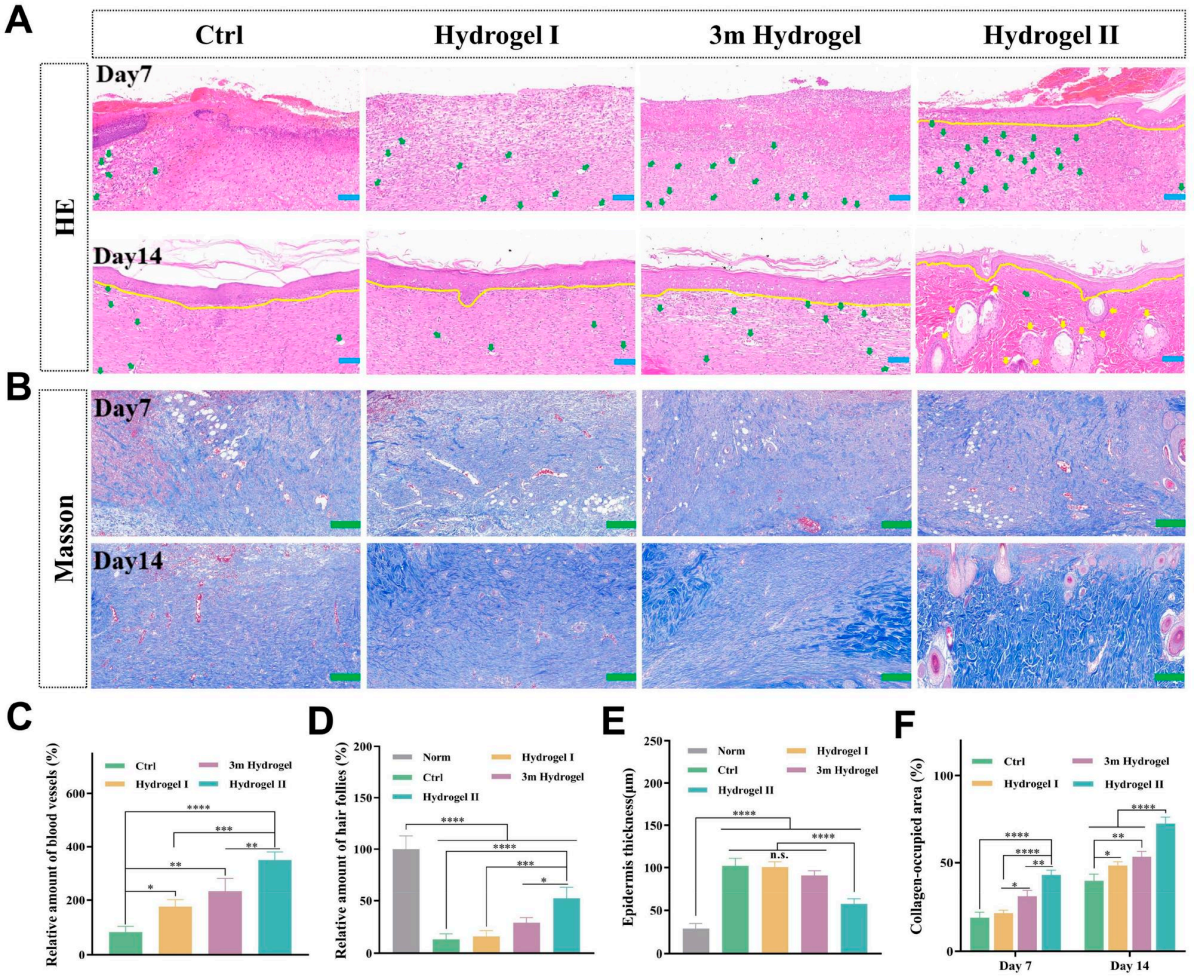
Histology of wound healing. (**A**) HE staining images of regenerated skin tissues, scale bar: 100 μm. (**B**) Masson's trichrome staining images of regenerated skin tissues, scale bar: 200 μm. (**C**) Relative amount of capillary under different treatments. (**D**) Hair follicle density of regenerated skin tissues in each group at Day 14. (**E**) Epithelial thickness of granulation tissues with different treatments at Day 14. (**F**) Quantitative analysis of collagen-occupied regions at Days 7 and 14. Note: Hydrogel I: the p(MMA-co-AM)/PVA hydrogel; hydrogel II: the p(MMA-co-AM)/PVA@PSO hydrogel. Data are shown as mean ± SD (*n* = 3), *P < 0.05, **P < 0.01, ***P < 0.005, ****P < 0.001, n.s.: no significance.

Histological evaluation revealed the treatment effect of the hydrogel on chronic diabetic wounds by clarifying the healing process of regenerated tissue. Histological analysis of the harvested skin tissues was performed by HE and Masson’s trichrome staining to obtain more details related to skin regeneration. As important functional skin structures, blood vessels and hair follicles were used to evaluate the integrity of tissue regeneration and the maturity of new skin structures [54, 55]. Through HE staining tissue sections, the neovascularization of the wound was observed under a microscope (Figure 8A). On Day 7, the density of new blood vessels (green arrows) in the hydrogel II group was significantly higher than that in the other three groups. And compared to the gauze group, there were more new blood vessels in the hydrogel I and commercial hydrogel groups. Moreover, the formation of hair follicles (yellow arrows) in each group was also analyzed. In the skin tissue sections on the 14th day, the number of hair follicles in the hydrogel II group was highest (Figure 8A and D). These results showed that the regenerated skin treated with hydrogel II had higher maturity.

Re-epithelialization is the thinning and maturation of epithelial structures and is an important stage of skin regeneration [5]. As shown in Figure 8A, the hydrogel II group exhibited partial epithelial coverage (yellow line) on the 7th day, whereas the other three groups exhibited no regenerated epithelial coverage. From 7 to 14 days, the epithelial thickness of the hydrogel II group gradually became thinner and denser. On Day 14, each group formed epithelial structures. The epithelium thickness of the hydrogel II group was the thinnest and similar to that of the normal group (Supplementary Figure S8), while the other groups had significantly thicker epithelium (Figure 8E), indicating the highest maturity of the regenerated skin in the hydrogel II group.

Collagen fiber is one of the key components of the ECM [56]. However, high levels of MMP-9 in chronic diabetic wounds often lead to excessive decomposition of the ECM (including collagen) [57]. Collagen deposition increased in all groups over time, with the hydrogel II group exhibiting the highest collagen deposition compared to the other groups (Figure 8B and F). This is the result of PSO inhibiting the activity of excessive MMP-9 and reducing ECM degradation, and more fibroblasts with better activity can also secrete more collagen [11, 58].

### Pre-healing factor analysis

#### p(MMA-co-AM)/PVA@PSO hydrogel promoted neovascularization and inhibited MMP-9

**Figure 9.**
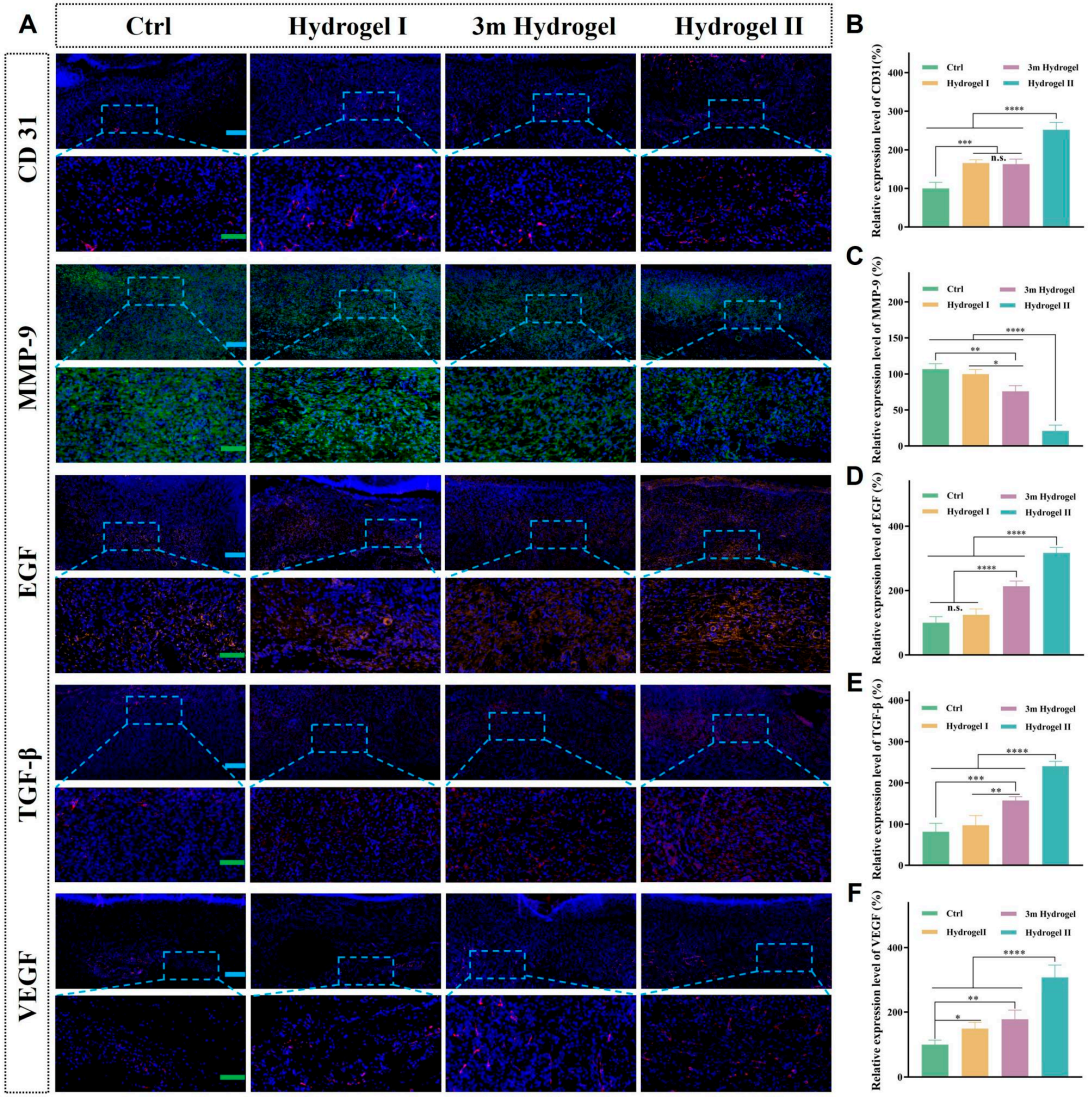
Immunofluorescence staining analysis of pre-healing factors. (**A**) Images of immune-fluorescence staining of CD31, MMP-9, EGF, TGF-β and VEGF were performed on the wounds of different groups, blue scale bar: 200 μm, green scale bar: 100 μm. (**B–F**) The quantitative analysis of the expression of CD31, MMP-9, EGF, TGF-β and VEGF. The data were normalized against the results of the ctrl group at Day 7, and the data of the ctrl group were defined as 100%. Note: Hydrogel I: the p(MMA-co-AM)/PVA hydrogel; hydrogel II: the p(MMA-co-AM)/PVA@PSO hydrogel. Data are shown as mean ± SD (*n* = 3), *P < 0.05, **P < 0.01, ***P < 0.005, ****P < 0.001, n.s.: no significance.

To further study the mechanisms by which the p(MMA-co-AM)/PVA@PSO hydrogel promoted diabetic wound healing, the typical pre-healing factors were analyzed. Angiogenesis plays an important role in tissue regeneration during wound healing [59]. Ischemia and hypoxia are also important causes of chronic non-healing wounds in patients with diabetes [60]. In addition, it has been reported that excessive secretion of MMP-9 in diabetic wounds injures cells related to wound repair (including fibroblasts, endothelial cells and keratinocytes), causing severe vascular damage [6, 7]. Therefore, immunofluorescence staining and RT-qPCR were used to further verify the levels of angiogenesis and MMP-9 in the regenerated tissues of different treatment groups. CD31, a typical biomarker for endothelial cells, was thus detected to identify the formation of neonatal capillary vessels [61]. The CD31-positive cells in the ctrl, hydrogel I and 3m hydrogel groups were relatively sparse, while dense red fluorescents were observed in the hydrogel II group (Figure 9A). The quantitative evaluation showed that the amount of vascularization in the hydrogel II group was significantly higher than that in the other three groups (Figure 9B). Moreover, in quantitative RT-qPCR analysis, the relative mRNA expression of CD31 in the hydrogel II group was significantly higher than that in the other three groups (Supplementary Figure S9). In addition, the mRNA level of CD31 of hydrogel I and 3m hydrogel was also higher than that of the gauze group. The results suggested that hydrogel II can stimulate micro-angiogenesis and promote skin angiogenesis. On Day 7, compared to the other three groups, a lower level of MMP-9 was detected in the hydrogel II group (Figure 9A and C). The relative mRNA expression of MMP-9 was the lowest in hydrogel II, but there was no significant difference among the other three groups (Supplementary Figure S9). This can be attributed to PSO’s role in reducing both the inflammatory response and oxidative stress, thus decreasing the expression of MMP-9 in diabetic wounds [13]. Therefore, the hydrogel can effectively release PSO in wounds, thereby inhibiting MMP-9 production and promoting vascularization.

#### p(MMA-co-AM)/PVA@PSO hydrogel increased EGF, TGF-β and VEGF expression in wounds

EGF, TGF-β and VEGF are three important growth factors involved in wound healing [62]. However, overproduction of MMPs in diabetic wounds leads to excessive breakdown of growth factors. Therefore, the expression of three growth factors related to wound healing was examined by immunofluorescence staining and RT-qPCR. As shown in Figure 9A, the three growth factors were significantly upregulated in the hydrogel II group compared to the other three groups. Through the quantitative analysis of three growth factors in different groups, the expression level of EGF in the hydrogel II group was 3.18, 2.54 and 1.50 times higher than that in the gauze, hydrogel I and commercial hydrogel groups, respectively (Figure 9D). The TGF-β level in the hydrogel II group was 2.95 times higher than that in the gauze group but 2.47 times and 1.53 times higher than that in the hydrogel I group and commercial hydrogel group, respectively (Figure 9E). Moreover, the hydrogel II group also showed 3.08 times, 2.05 times and 1.73 times more than the gauze group, hydrogel I group and commercial hydrogel treatment group for VEGF expression level (Figure 9F). Simultaneously, further quantitative RT-qPCR analysis showed that the relative mRNA expression of the three growth factors in the hydrogel II group was also the highest (Supplementary Figure S9). This can be attributed to the release of PSO from hydrogel II, which inhibits MMPs, consequently reducing the degradation of growth factors and cell damage [10]. Additionally, studies have shown that PSO can stabilize related growth factors and increase the utilization of growth factors in the body [11, 13, 63]. Moreover, the porous structure of double-network hydrogel simulates ECM and creates a moist and suitable wound environment, fostering optimal conditions for cell growth [20, 64]. More fibroblasts and more mature granulation tissue also secreted more EGF, TGF-β and VEGF [52]. Therefore, hydrogel II significantly increased the level of growth factors in diabetic wounds, thereby promoting the healing of the diabetic wounds.

## Conclusion

In summary, this study utilized the HIPE template method to fabricate a novel double-network porous hydrogel, serving as a carrier for the delivery of PSO drugs and successfully applying it to facilitate the healing of diabetic wounds. The p(MMA-co-AM)/PVA@PSO hydrogel showed remarkable material properties and biological characteristics. The interconnected pore structure constructed by HIPE increased the hydrogel’s porosity, thereby enhancing the drug loading and water absorption performance of the hydrogel. The double-network improved the mechanical properties of the hydrogel. In addition, further study results indicated that the hydrogel could effectively release PSO, leading to the inhibition of MMP-9 in diabetic wounds, an increase in growth factor secretion, promotion of neovascularization, collagen deposition and re-epithelialization, thus accelerating the healing of diabetic wounds. Therefore, this work presented a new and promising porous hydrogel wound dressing for diabetic wound healing and proposed a viable therapeutic approach to promoting diabetic wound healing early. Simultaneously, it contributed some theoretical foundations and practical experiences for the application of the HIPE technique in the field of biomedicine.

## Supplementary Material

rbae024_Supplementary_Data
