# Supplementary material for: A double-network porous hydrogel based on high internal phase emulsions as a vehicle for potassium sucrose octasulfate delivery accelerates diabetic wound healing
Source: Regen Biomater. 2024 Mar 12;11:rbae024. doi: 10.1093/rb/rbae024 (PMC11018543; doi:10.1093/rb/rbae024)
Supplement: rbae024_Supplementary_Data [file rbae024_supplementary_data.docx]

**Supporting Information**

**A double-network porous hydrogel based on high internal phase emulsions as a vehicle for potassium sucrose octasulfate delivery accelerates diabetic wound healing**

Zhiwei Wang ^1#^, Lingshun Sun ^2#^, Weixing Wang ^1^, Zheng Wang ^1^, Ge Shi ^1^, Honglian Dai ^2*^, and Aixi Yu ^1*^

^1^ Department of Orthopedics Trauma and Microsurgery, Zhongnan Hospital of Wuhan University, Wuhan, 430070, China

^2^ State Key Laboratory of Advanced Technology for Materials Synthesis and Processing, Biomedical Materials and Engineering Research Center of Hubei Province, Wuhan University of Technology, Wuhan, 430070, China

^*^ Corresponding authors:

Honglian Dai. Address: State Key Laboratory of Advanced Technology for Materials Synthesis and Processing, Biomedical Materials and Engineering Research Center of Hubei Province, Wuhan University of Technology, Wuhan, 430070, China.

E-mail: daihonglian@whut.edu.cn

Aixi Yu. Address: Department of Orthopedics Trauma and Microsurgery, Zhongnan Hospital of Wuhan University, Wuhan, 430070, China.

E-mail: yuaixi@whu.edu.cn

^#^ Zhiwei Wang and Lingshun Sun contributed equally to this work.

1. **Experimental section**
   1. **Thermal stability analysis of hydrogels**

Differential scanning calorimetry (DSC) and thermogravimetric (TG) analysis of the hydrogels were performed on a simultaneous thermal analyzer (STA2500, NETZSCH, Germany). The heating rate used was 10°C/min and the measurements were performed in N_2_ atmosphere.

**1.2. Mechanical property testing of hydrogels**

Mechanical experiments were performed on the electronic universal testing machine (UTM2503, Shenzhen Suns Technology Stock Co., Ltd., China) at room temperature. The tensile properties of the hydrogel were obtained by stretching long strip samples (50 mm in length, 10 mm in width, and 1mm in height) at a strain speed of 5 mm/min. The cylindrical samples (12 mm in diameter and 10 mm in height) were used for the compressive experiments at a compression velocity of 5 mm/min. The fatigue resistance of the hydrogel was measured by cyclic loading-unloading tests. Specifically, the cylindrical sample was repeatedly compressed at a compression velocity of 5 mm/min when the maximum strain was kept at 50%.

**1.3. Swelling behavior of hydrogels**

The cylindrical hydrogels of the same volume (12 mm in diameter and 10mm in height) were cut, weighed, and measured, and then immersed in PBS solution (37 °C, pH = 7.4). The weight and diameter of the swelling hydrogels were measured every 12 hours, and the change rate was calculated according to the following formula:

Weight and Diameter Change Ratio (%) = [(Wa － Wi) / Wi] × 100

Wa represents the weight or diameter of the hydrogels after soaking, and Wi represents the initial weight or diameter of the hydrogel.

**1.4. Endothelial cell tube formation experiment**

The hydrogel extract was co-cultured with HUVECs in a 6-well plate for 24 h, and then the digested cells were prepared into three groups of cell suspensions. The HUVECs density was adjusted to 2 × 105 cells/mL. The 24-well plate and the gun head of 1 mL were pre-frozen in a refrigerator at -20 °C, and Matrigel (BD, USA) was thawed overnight at 4 °C. The Matrigel matrix glue was added to the 24-well plate at a ratio of 250 µL/well, then the orifice plate was placed in a cell incubator at 37 °C for 1 h to solidify, then 500 µL cell suspension was added to each hole, and the culture plate was placed in an incubator to form a tube. After being cultured for 24 h, the cytoskeletons were stained for easier visualization of morphology, and tube formation was observed under a light microscope (Soptop ICX41, Sunny Optical Technology Co., Ltd.), and the average tube formation value was calculated by Image J v1.8.0 software.

- 1. **Cell proliferation and migration in a simulated diabetic wound environment**

According to the methods in the literature [1], glucose and MMP-9 were added to simulate the high levels of glucose and MMP-9 in diabetic wounds to evaluate the effects of different hydrogels on the proliferation of fibroblasts and endothelial cells. In short, the NIH3T3 cells and bEnd.3 cells were cultured in complete cell culture medium containing 10 mg/mL glucose, 50 nM MMP-9, and 0.1 g/mL hydrogel for 24 h. Then the cell proliferation was detected by an EdU kit. The effect of hydrogels on cell migration was evaluated by the cell scratch test. After NIH3T3 cells were cultured for 24 h, 10 mg/mL glucose and 50nm MMP-9 were added, and the cell layer was scraped with a 200 μL transfer tube tip, and then the cells were treated with hydrogel extracts. Scratch healing was observed and photographed at 0, 12, 24, 48, and 60 hours after treatment.

- 1. **PCR Assay**

Skin tissues were collected on day 7 after the operation, and the expressions of CD31, MMP-9, EGF, TGF-β, and VEGF were detected by RT-qPCR. According to the manufacturer’s protocol, total RNA was extracted from skin tissues with TRIzol reagent (Servicebio, China) and ran on an RT-qPCR system (KUBO, China). The primer sequences used in RT-qPCR analysis are shown in Table S1.

**Table S1. Primer sequences for RT-qPCR.**

| Primer | Forward | Reverse |
| --- | --- | --- |
| GAPDH | AGACAGCCGCATCTTCTTGT | CTTGCCGTGGGTAGAGTCAT |
| CD31 | CGAAATCTAGGCCTCAGCAC | CACCGTCAGCTGGTACTCAA |
| MMP-9 | TTTCTTCTTCTCTGGGCGCAA | TTTCCAGATACGTTCCCGGC |
| EGF | CGAACGATGCAAACAGAGAA | TACAGCCGTGATTCTGCAAG |
| TGF-β | GACCGCAACAACGCAATCTA | CGTGTTGCTCCACAGTTGAC |
| VEGF | GCCTCAGGACATGGCACTAT | GAGGAGGAGGAGCCATTACC |

1. **Supporting Tables and Figures**


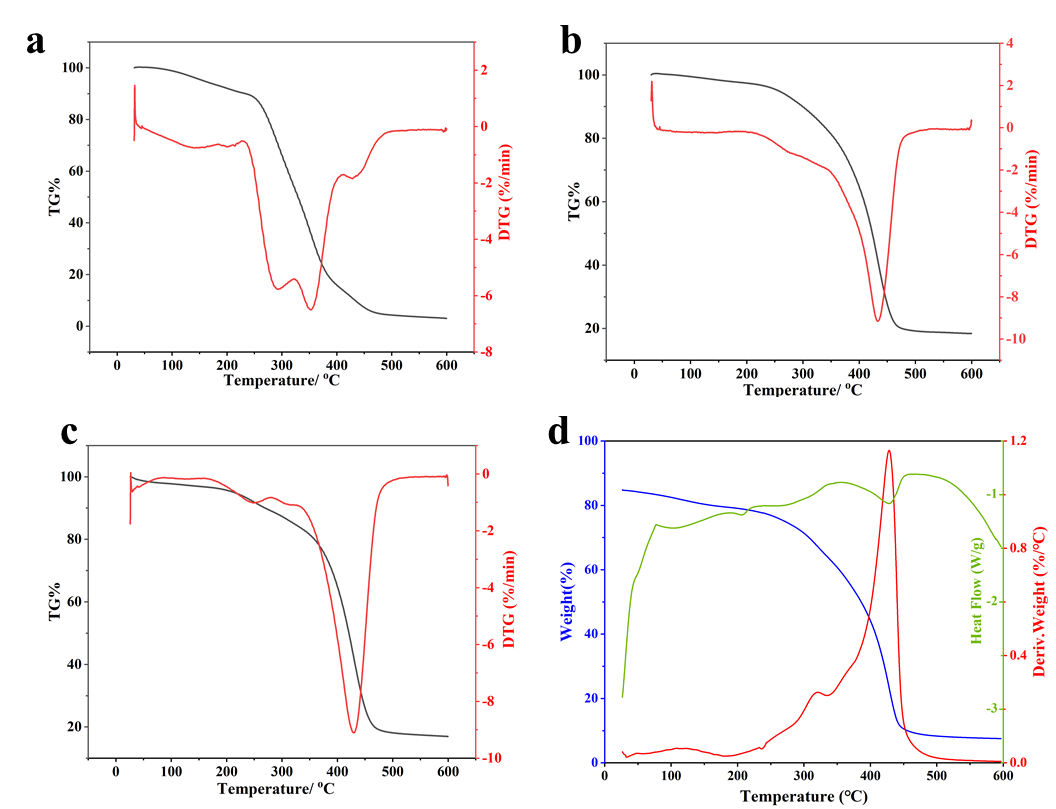


**Figure S1.** Thermal stability analysis of hydrogels. a), b), and c) The TG-DTG curves of PVA, p(MMA-co-AM) and p(MMA-co-AM)/PVA hydrogels, respectively. d) The DSC-TG curve of p(MMA-co-AM)/PVA hydrogel.

**
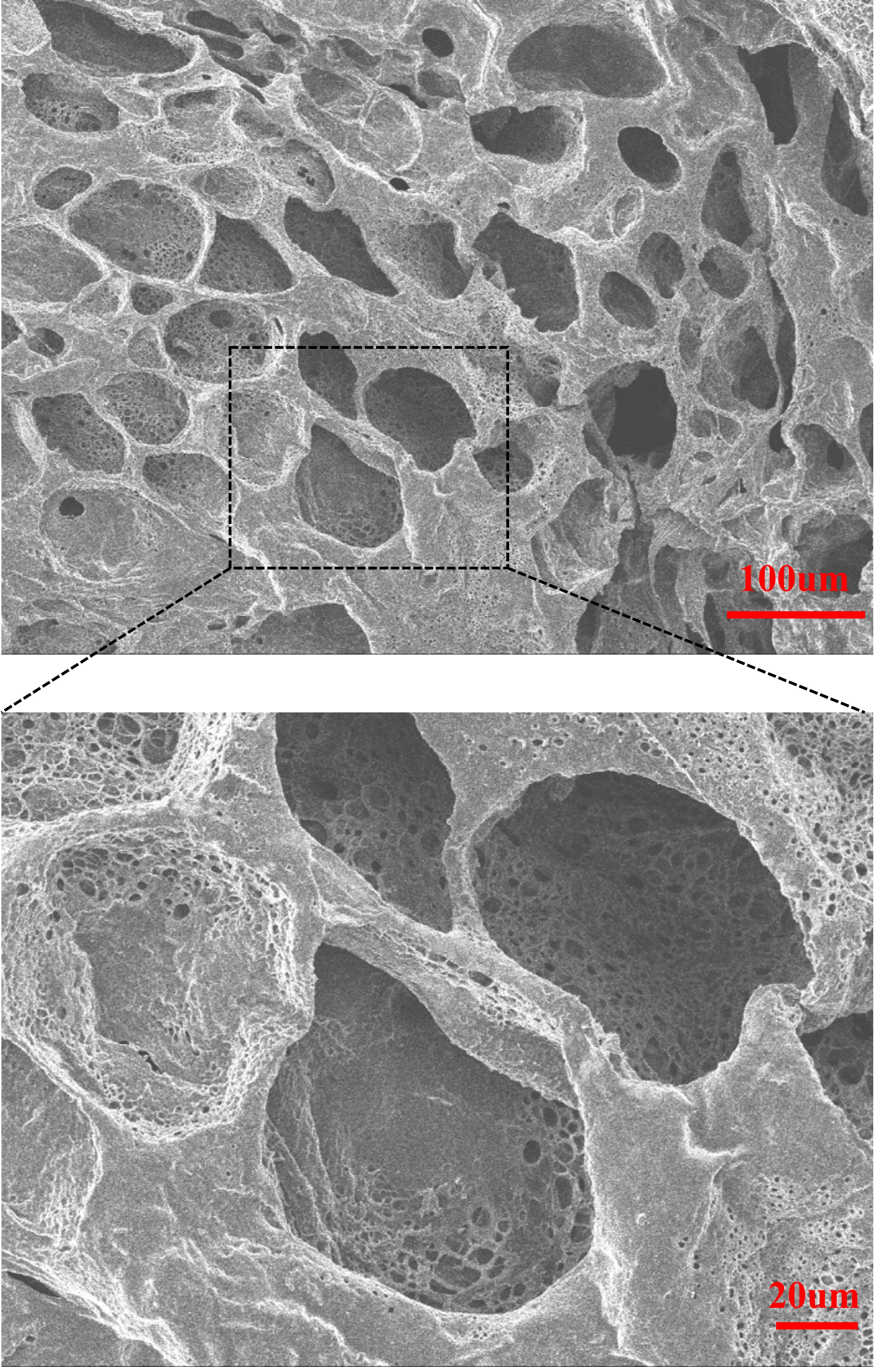
**

**Figure S2.** The SEM images of p(MMA-co-AM).


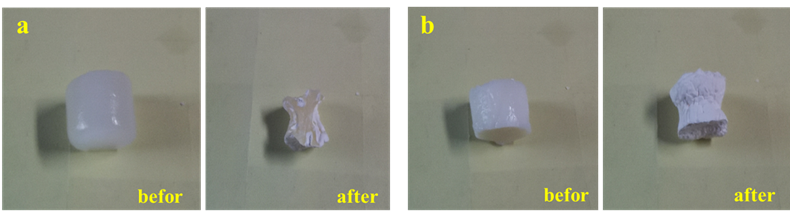


**Figure S3.** The morphological change of the PVA hydrogel and the p(MMA-co-AM)/PVA hydrogel before and after drying. a) The morphological change of the PVA hydrogel before and after drying, b) The morphological change of the p(MMA-co-AM)/PVA hydrogel before and after drying.


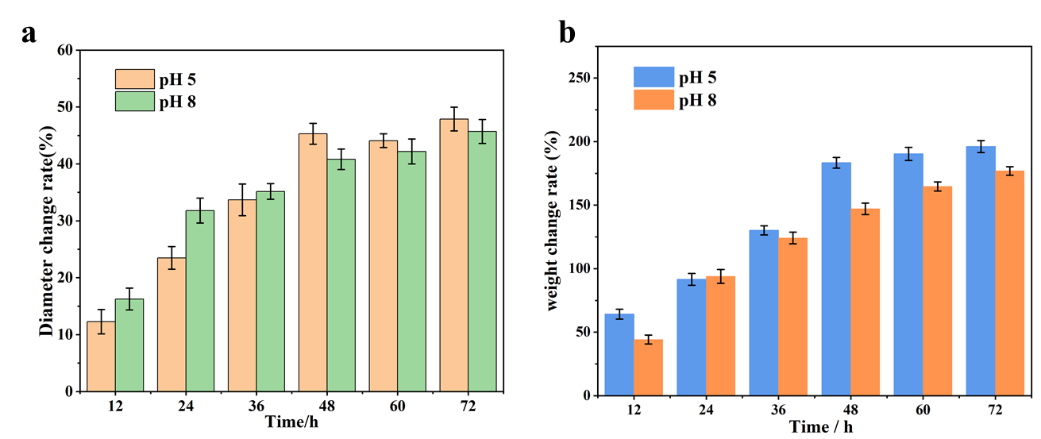


**Figure S4.** Swelling properties of P8-H95 double-network porous hydrogels at pH 5 and pH 8.


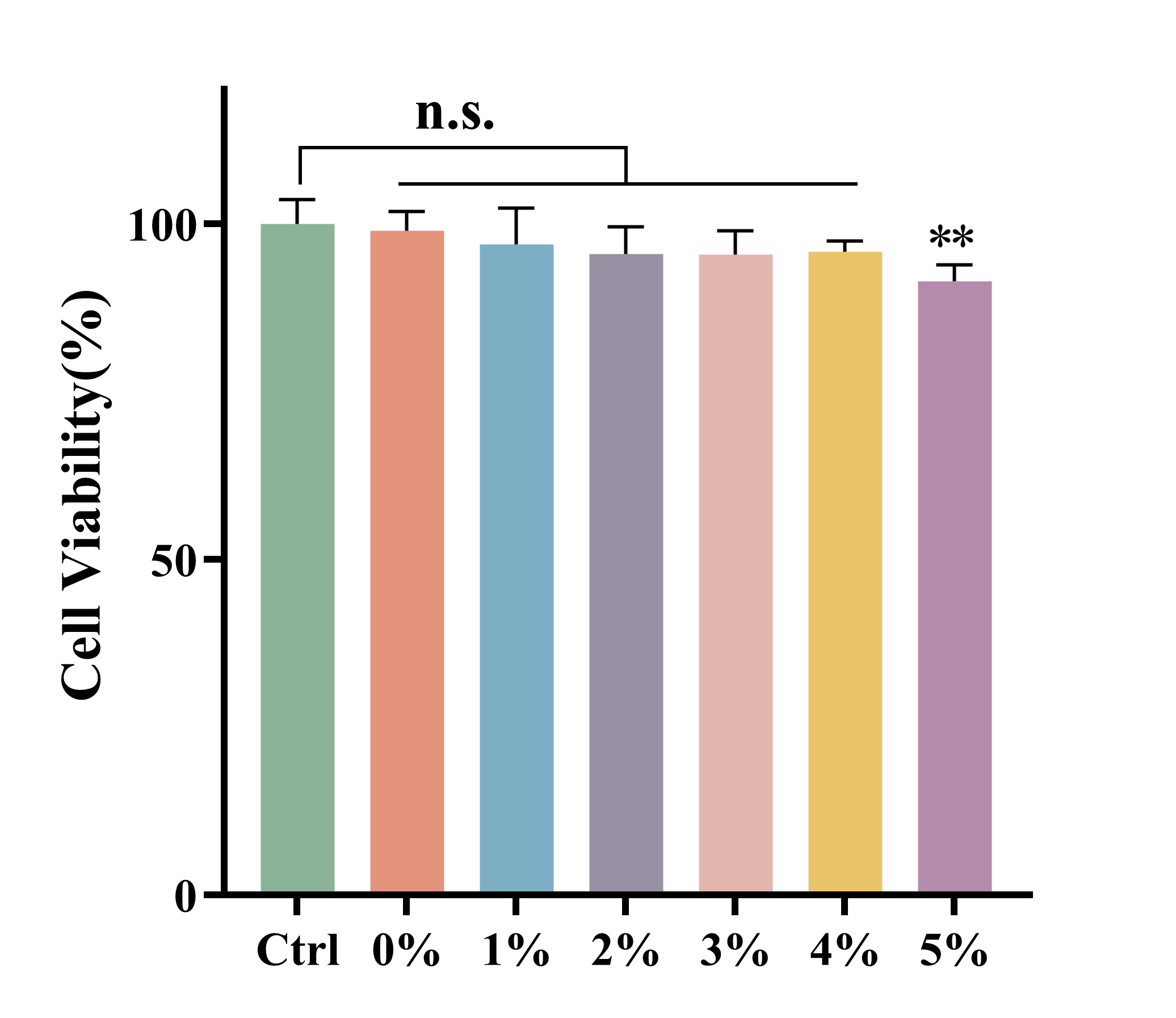


**Figure S5.** Effects of the p(MMA-co-AM)/PVA@PSO hydrogels with different PSO concentrations on the activity of NIH3T3 cells.

**
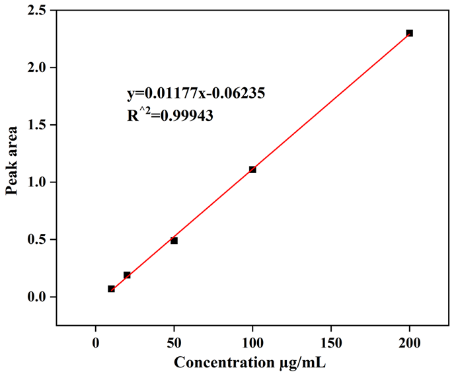
**

**Figure S6.** Linear equation for quantitative detection of PSO release.

**Table S2.** The linear range, regression equation, correlation coefficient, and limit of detection of PSO

| **Analyte** | **R^2^** | **Regression equation** | **Linear range (µg mL^-1^)** | **LOD (µg mL^-1^)** |
| --- | --- | --- | --- | --- |
| **PSO** | 0.99943 | Y=0.01177X-0.06235 | 10-200 µg | 0.1 |

**Table S3.** Recoveries and relative standard deviations of PSO from PBS (n=3)

| **Analyte** | **Spiked addition (µg mL^-1^)** | **Recovery (n=3, %)** | **RSD (n=3, %)** |
| --- | --- | --- | --- |
| **PSO** | 0 | - | - |
|  | 10 | 95.3 | 0.8 |
|  | 20 | 97.4 | 0.6 |
|  | 50 | 102.6 | 0.7 |
|  | 100 | 90.3 | 1.3 |
|  | 200 | 97.2 | 2.1 |


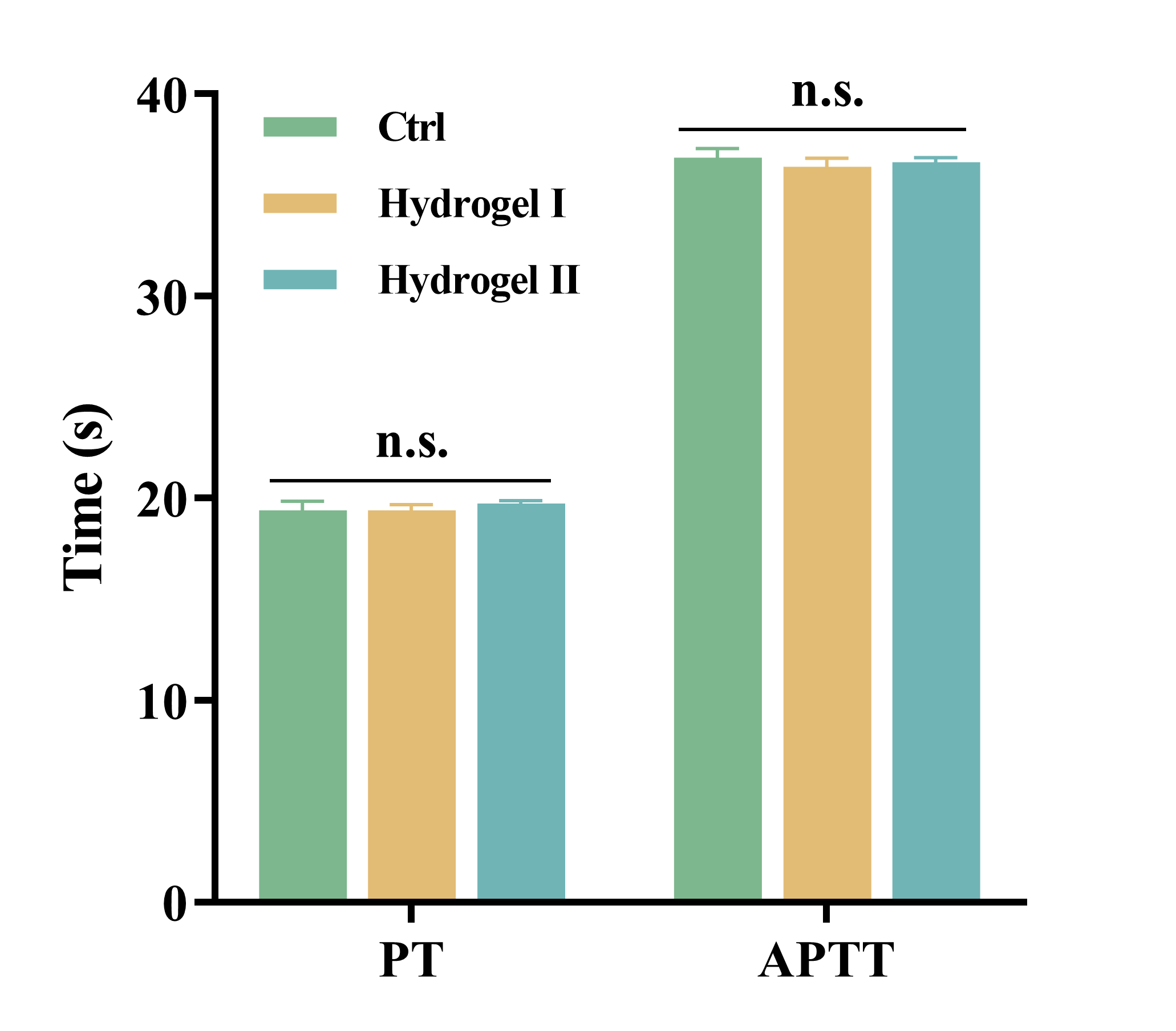


**Figure S7.** The in-vitro coagulation assay of hydrogels (PT and APTT). Note: Hydrogel I: the p(MMA-co-AM)/PVA hydrogel; Hydrogel II: the p(MMA-co-AM) /PVA@PSO hydrogel. Data are shown as mean ± SD (n = 3), n.s.: no significance.


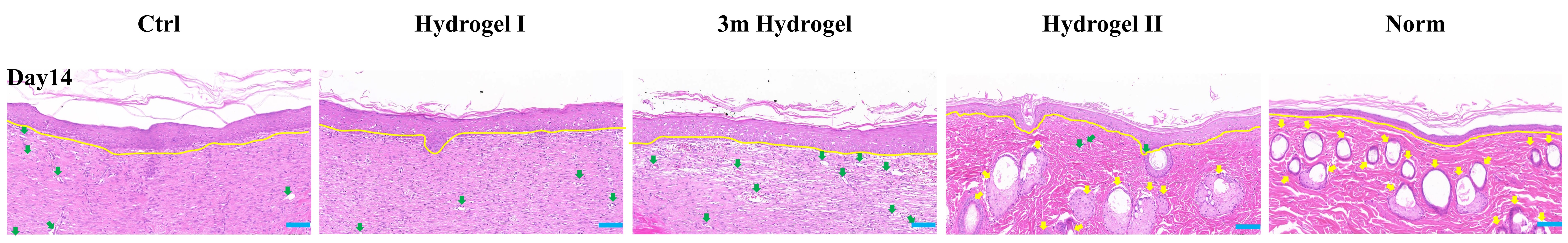


**Figure S8.** HE staining images of normal skin tissue in diabetic rats and regenerated skin tissue in different treatment groups, scale bar: 100 μm. Note: Norm: normal, Ctrl: control, Hydrogel I: the p(MMA-co-AM)/PVA hydrogel, Hydrogel II: the p(MMA-co-AM)/PVA@PSO hydrogel, 3m Hydrogel: 3m Tegaderm hydrogel.


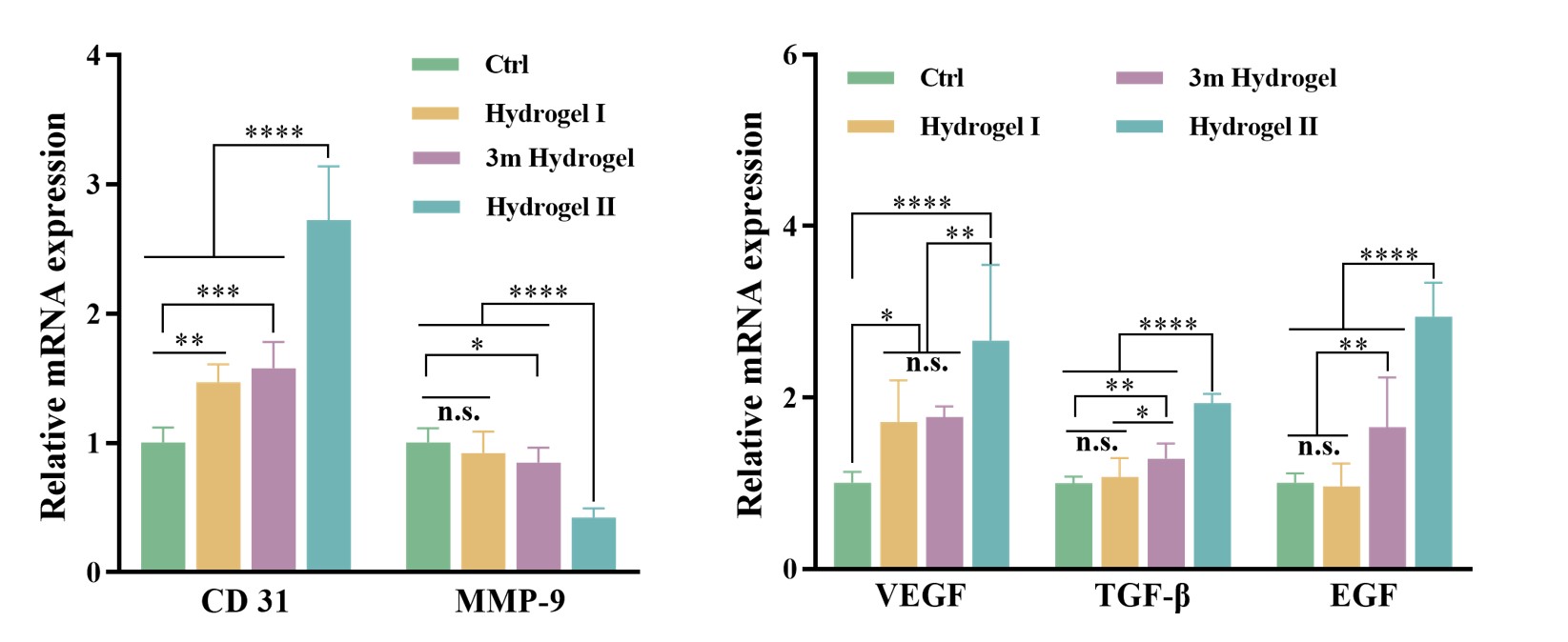


**Figure S9.** Relative mRNA expression of CD31, MMP-9, EGF, TGF-β, and VEGF in repaired wound tissues 7 days after different treatments. Note: Hydrogel I: the p(MMA-co-AM)/PVA hydrogel; Hydrogel II: the p(MMA-co-AM) /PVA@PSO hydrogel. Data are shown as mean ± SD (n = 3), *p < 0.05, **p < 0.01, ***p < 0.005, ****p < 0.001, n.s.: no significance.

**Reference**

1. Zhou W, Duan Z, Zhao J, Fu R, Zhu C, Fan D. Glucose and MMP-9 dual-responsive hydrogel with temperature sensitive self-adaptive shape and controlled drug release accelerates diabetic wound healing. *Bioact Mater* 2022;17:1-17.
